# Inter-assay variation and reproducibility of progesterone measurements during ovarian stimulation for IVF

**DOI:** 10.1371/journal.pone.0206098

**Published:** 2018-11-01

**Authors:** Barbara Lawrenz, Junard Sibal, Nicolas Garrido, Emmanuel Abu, Alliza Jean, Laura Melado, Human M. Fatemi

**Affiliations:** 1 IVIRMA Middle-East Fertility Clinic, Abu Dhabi, United Arab Emirates; 2 Obstetrical Department, Women´s University Hospital Tuebingen, Tuebingen, Germany; 3 IVIRMA Middle-East Fertility Clinic, Laboratory, Abu Dhabi, United Arab Emirates; 4 IVI Foundation, IVI Valencia, Valencia, Spain; Jagiellonian University Medical College, UNITED STATES

## Abstract

In recent years there is increasing evidence that elevated progesterone levels during ovarian stimulation for IVF / ICSI have a negative impact on the ART-outcome. However, different progesterone assays were used in the previous studies and different assays might produce varying results. This retrospective study evaluated the reproducibility and reliability of different progesterone assays with a special focus on progesterone levels below 1.5 ng/ml, as this range is crucial for early detection of progesterone rise during ovarian stimulation for IVF. A total of 413 blood samples were categorized in different progesterone ranges and whether they were retrieved on the day of final oocyte maturation and the results were compared regarding their reproducibility and reliability. To compare the reproducibility between the different progesterone assays, the Intraclass Correlation Coefficient (ICC) was calculated and interpretation of the ICC results was done according to Cicchetti, ranging from poor to excellent. The correlation of the assays was excellent when all samples were compared including samples retrieved on day of final oocyte maturation, however in the ranges of progesterone levels 1.0 ng/ml to < 1.5 ng/ml, 0.8 ng/ml to < 1.0 ng/ml and < 0.8 ng/ml, the ICC varied between poor and excellent. The assays “gen III” and “Architect” showed an excellent reproducibility of progesterone results throughout all ranges of progesterone levels.

This analysis demonstrates, that different progesterone assays have a limited reproducibility and that the results depend on the assay used and the range of progesterone level. This fact leads to two important conclusions. Firstly the limited reproducibility might lead to substantially different treatment decisions in ovarian stimulation treatment for IVF and secondly critical interpretation of thresholds, provided by meta-analysis, is crucial despite the risk that the so far gained clinical experience might become irrelevant and has to be adjusted to the results, obtained by each assay.

## Introduction

In recent years, progesterone elevation during the late follicular phase of ovarian stimulation for In-vitro-fertilisation (IVF)–treatment and its impact on the pregnancy rates is a matter of intense research and ongoing debate.

Many studies confirmed the negative impact on the pregnancy rate in fresh embryo-transfer cycles attributed to progesterone elevation on the day of final oocyte maturation which results in endometrial advancement and subsequent asynchrony between the endometrium and the embryo. Lately, several studies also clearly demonstrated a reduction in the number of top quality embryos in patients with elevated progesterone levels [1] and a significant reduction in cumulative pregnancy rates [2]. The initial studies demonstrated significantly reduced pregnancy rates with arbitrarily chosen progesterone levels above a threshold of 0.9 ng/ml and 1.1 ng/ml [3,4], however, subsequent studies used different cut-off-levels to define progesterone elevation during stimulated cycles. The various cut-off-levels in these studies ranged from 0.8 to 2.0 ng/ml [5–10].

The most extensive data are summarized in the meta-analysis of Venetis et al. [11], which demonstrated a significant decrease in ongoing pregnancy rates with serum progesterone levels above 1.5 ng/ml on the day of final oocyte maturation. This meta-analysis comprises more than 60.000 cycles from 63 studies, published between 1990 and 2012. Chronologically, the first study included, is the publication of Edelstein et al. in 1990 [12] and the most recent study included was published by Xu et al. in 2012 [13]. The study inclusion criteria, used for this meta-analysis are described in detail [11]. Interestingly the assays, used for progesterone measurement, were neither part of the inclusion, nor of the exclusion criteria. Due to the timespan of 22 years between the first and the final studies included, different techniques and progesterone assays have been used for progesterone measurement. In the study of Edelstein et al. progesterone measurement was performed with “Commercially available RIA (radio immuno assays) kits (Pantex, Santa Monica, CA) to determine E2 and P” and in the study of Xu et al. “microparticle enzyme immunoassay (Axsym System, Advia Centaur; Siemens)”, was the preferred assay with many different assays used in the intervening studies.

It is clear that different progesterone-assays have been applied in the included studies and as it was shown previously, different assays will deliver different results, despite measuring the same hormonal parameter and differences in the inter-assay performance could contribute to heterogeneous results [14].

The aim of this study is to compare different assays for progesterone evaluation and evaluate the reproducibility of the results.

## Material and methods

In this observational retrospective study, performed between June and September 2017, data from blood samples from patients either planned for or actually undergoing ovarian stimulation for IVF / ICSI due to primary or secondary infertility were analysed with 3 different progesterone assays as a clinical routine between June and September 2017, as the assay ELECSYS generation II by Roche, which was routinely used for progesterone measurement in our clinic, was announced to be taken from the market. The different assays were: ELECSYS generation II by Roche (gen II), ELECSYS generation III by Roche (gen III) and „Architect”by Abbott (Architect).

The blood which was taken as a routine procedure from patients under treatment, was centrifuged for 10 minutes at 4000 rpm (revolution per minute) and the supernatant was retrieved. Half of the serum was used for hormonal evaluation on the day of blood retrieval for clinical decisions regarding the ongoing ovarian stimulation treatment and the other half of the blood was frozen at– 21°C. This approach is clinical routine in our centre with every blood sample in case the blood test may have to be re-run.

For the progesterone measurement for this analysis with the assays „gen II“, „gen III”and „Architect“, the samples were thawed by keeping them for maximum 20 minutes at room temperature (approximately 20°C—24°C) and analysed the same day with the same batch of reagents.

ELECSYS progesterone generation II assay is an electrochemiluminescence immunoassay (ECLISA) which uses mouse monoclonal antibodies. The measuring range is 0.095–191 nmol/L or 0.030–60 ng/ml. For detection of analytical specificity, cross-reactivities towards other hormones were used with a maximum cross-reactivity of 0.858% towards 5α-Pregnen-3β-ol-20-on and the minimum cross-reactivity of 0.002% towards Androstendiol and Ethisterone [15].

In the assay ELECSYS progesterone generation III, the mouse monoclonal antibodies have been replaced with sheep monoclonal antibodies due to their higher specificity towards progesterone. The measuring range is 0.159–191 nmol/L or 0.05–60 ng/ml. For detection of analytical specificity, cross-reactivities towards other hormones were used with a maximum cross-reactivity of 3.93% towards 11-Deoxycorticosterone and the minimum cross-reactivity of 0.001% towards Danazol [16].

The „Architect”assay by Abbott is a one-step chemiluminescent microparticle immunoassay using sheep monoclonal antibodies. The analytical sensitivity was calculated to be better than 0.1 ng/mL. For detection of analytical specificity, cross-reactivities towards other hormones were used with a maximum cross-reactivity of 4.6% towards Corticosterone and the minimum cross-reactivity of 0.1% towards Danazol, Medroxyprogesterone, 19-Nor-4-androsten-3.17-dione, Norethindrone, 19-Nortestosterone and Pregnenolone [17].

The progesterone levels obtained by the different assays were stratified according to two approaches: firstly, whether the blood sample was taken in preparation for or during ovarian stimulation or secondly, on the day of final oocyte maturation, as well as according to different ranges of the progesterone levels, measured with ELECSYS progesterone generation II assay, into the ranges: < 0.8 ng/ml; 0.8 - < 1.0 ng/ml; 1.0 - < 1.5 ng/ml; 1.5 ng/ml and above.

### Ethical approval

Due to the retrospective nature of this study, the ethical committee waived the need for consents. This analysis was approved by the ethics committee of IVI Middle East Fertillity Clinic Abu Dhabi, UAE (REFA014/2017).

### Statistical analysis

Comparison between the results of progesterone levels, measured by “gen II” versus “gen III” versus “Architect” was performed to evaluate each assays´ performance, diagnostic ability and overall reproducibility. This analysis was conducted by first plotting data on both measurements, and also conducting Reliability tests by means of calculations of the Intraclass Correlation Coefficients (ICC) and their corresponding 95%CI of both single and average measures.

The ICC for single measures is an index for the reliability of a specific assay, whereas the ICC for average measures represents the reliability that would be achieved when using the average of two assays to measure the level of progesterone. In our analysis, the “single measures ICC” is the appropriate coefficient to quantify the reliability.

For the calculation of the ICC a two-way mixed effects model was used where individual effects are random and assay effects are fixed and consistent. This model was applied as the intention of this analysis is to generalize the herein described reliability results to any raters who possess the same characteristics as the selected raters in the reliability study [18]. SPSS software (version 23) was used for all statistical analyses, and p values below 0.05 were used to indicate statistically significant differences.

## Results and discussion

Data from a total of 413 blood samples, obtained from 119 patients who were about to start or were under ovarian stimulation between June and September 2017. The age of the patients ranged from 23 to 48 years with a mean age of 35.94 years and the mean Body Mass Index (BMI) was 26.21 kg/m^2^ (range 14.34–38.22 kg/m^2^). The mean number of progesterone measurements per patient was 2.39 with a range of 1 to 6 samples. Measurement on one system or another depended on reagents and platform availability.

Data were available from 413 samples which were measured with „gen II”and „gen III”and from 121 samples close to or directly on the day of final oocyte maturation, which were run with all 3 progesterone assays. Out of the 121 samples, 72 samples were obtained from patients directly on the day of final oocyte maturation and 49 samples from patients close to the day of final oocyte maturation (trigger day minus 1–2 days). In 73 cases out of all measurements, progesterone levels were under the detection limit of the assay used. This affected assay “gen3” in 72 cases and assay “Architect” in one case. No result was under the detection limit with assay “gen II”.

After stratifying according to the results of the progesterone measurement with „gen II“, 34 samples were in the group with progesterone levels ≥ 1.5 ng/ml, 45 samples in the group 1.0 ng/ml to < 1.5 ng/ml, 30 samples in the group 0.8 ng/ml to < 1.0 ng/ml and 304 samples < 0.8 ng/ml. As progesterone levels on the day of final oocyte maturation are crucial for the decision for or against cryopreservation of the embryos, the samples obtained either close to the trigger day or directly on the trigger day were also stratified accordingly and the latter ones underwent ICC analysis.

For the aforesaid stratification, results under the detection limit (< 0.05 ng/ml) were treated as “0.00” because precise values could not be acquired. The means, 95% confidence intervals (95%CI) and standard deviations (SD) are presented in Table 1. It has to be noted, that for descriptive purposes the results below the detection limits (< 0.05 ng/ml) were included and calculated as “0.00” as already mentioned. A summary of all progesterone measurements is presented in the supplementary S4 File, distinguished in all samples, stratified according to the progesterone levels and samples obtained from the day of final oocyte maturation.

**Table 1:** Means, 95% confidence intervalls (95%CI) and standard deviations (SD) for the progesterone results, results below the detection range are calculated as „0.00“.

Type of sample	Number of samples	Mean (ng/ml)	95% Confidence Intervall (95%CI)	Standard-deviation (SD)
**All samples**				
„gen II”	413	1.13	0.78–1.47	3.55
„gen III”	413	0.76	0.47–1.05	2.95
„Architect“	121	0.75	0.67–0.83	0.46
**Samples corresponding to „gen II”≥ 1.5 ng/ml**				
„gen II”	34	7.42	3.73–11.11	10.57
„gen III”	34	6.44	3.47–9.40	8.50
„Architect“	14	1.57	1.31–1.84	0.46
**Samples corresponding to „gen II”1.0 - < 1.5 ng/ml**				
„gen II”	45	1.22	1.17–1.26	0.13
„gen III”	45	0.68	0.60–0.77	0.27
„Architect”	26	1.02	0.89–1.14	0.31
**Samples corresponding to „gen II”0.8 - < 1.0 ng/ml**				
„gen II”	30	0.89	0.87–0.91	0.57
„gen III“	30	0.51	0.42–0.60	0.24
„Architect”	9	0.76	0.58–0.93	0.22
**Samples corresponding to „gen II”< 0.8 ng/ml**				
„gen II”	304	0.44	0.42–0.46	0.18
„gen III”	304	0.16	0.15–0.18	0.16
„Architect”	70	0.49	0.43–0.54	0.23
**Samples on the trigger day and close to the trigger day**				
„gen II”	121	0.86	0.77–0.94	0.49
„gen III”	121	0.46	0.39–0.54	0.42
„Architect”	121	0.75	0.67–0.84	0.46
**Samples on the trigger day**				
„gen II”	72	0.89	0.78–1.01	0.49
„gen III”	72	0.48	0.38–0.57	0.40
„Architect”	72	0.77	0.67–0.87	0.43
**Samples close to the trigger day**				
„gen II”	49	0.79	0.65–0.94	0.49
„gen III”	49	0.44	0.31–0.56	0.44
„Architect”	49	0.74	0.59–0.88	0.51

To compare the exactness between the different progesterone assays, the Intraclass Correlation Coefficient (ICC) was calculated and interpretation of the ICC results was done according to Cicchetti [19] et al. as follows: Less than 0.40—poor; Between 0.40 and 0.59—fair; Between 0.60 and 0.74—good; Between 0.75 and 1.00—excellent [18]. The ICC for single measures were 0.851 (95%CI: 0.771–0.904) for „gen II”vs „gen III“, 0.803 (95%CI: 0.702–0.872) for „gen II”vs „Architect”and 0.955 (95%CI: 0.929–0.971) for „gen III”vs „Architect“.

**Fig 1:**
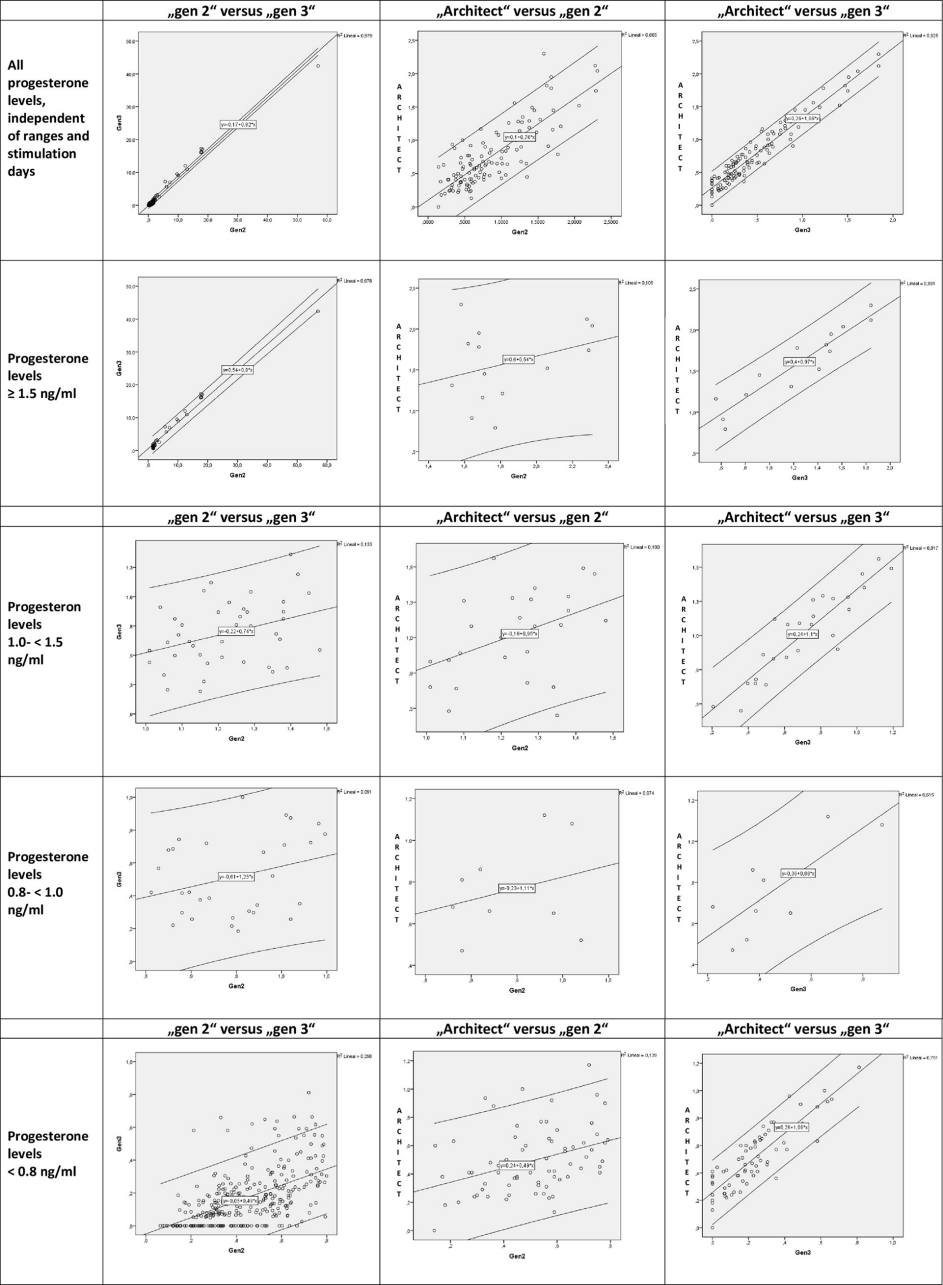
Reproducibility of the progesterone assays „gen 2“, „gen 3”and „Architect“. Progesterone levels are represented as scatter plots and with the confidence interval 95% (outer lines). X- and Y-axis presenting the progesterone levels in ng/ml in all diagrams.

In the group of progesterone-levels 1.0 - < 1.5 ng/ml, 42 samples were measured with „gen II”and „gen III”and 26 samples were measured with all 3 progesterone assays. The mean P4-levels (ng/ml), measured with „gen II“, „gen III”and „Architect”were 1.21, 0.66 and 0.99, respectively. The ICC for single measures were 0.288 (95%CI: 0.014–0.542) for „gen II”vs „gen III“, 0.315 (95%CI: 0.075–0.621) for „gen II”vs „Architect”and 0.887 (95%CI: 0.764–0.948) for „gen III”vs „Architect“. The correlation of the assays was excellent when all samples were compared as well as on the trigger day, however in all mentioned ranges of progesterone levels (> 1.5 ng/ml; 1.0 ng/ml to < 1.5 ng/ml; 0.8 ng/ml to < 1.0 ng/ml and < 0.8 ng/ml) the ICC varied between poor and excellent. It has to be noted, that in the above presented ICC calculation, progesterone results below the detection range were treated as 0.00. As a consequence of this decision, a zero-variance in some observations could be introduced which could potentially pose a problem for ICC estimation. Therefore, ICC calculations were repeated after deletion of the results <0.05 ng/ml and it was seen, that the values of ICC changed slightly, however the interpretation according to Cicchetti et al. [19] did not and thus the overall conclusions remain the same. Table 2 summarizes the number of samples for each progesterone range group and the ICC and Fig 1 represents the results as scatter plots. S1 Table summarizes the data of the re-analysis, run after the removal of progesterone levels below the detection range of the progesterone assays and is available as supplementary file.

**Table 2:** Reproducibility of the progesterone assays „gen 2“, „gen 3”and „Architect”according to Intraclass Correlation Coefficient (ICC) and interpretation according to Cicchetti et al. [19].

	Comparison between	No of cases	ICC single measures	Inter-pretation	ICC average measures	Inter-pretation
**all progesterone levels**	gen 2 vs gen 3	413	0.973 95%CI: 0.968–0.979	Excellent	0.986 95%CI: 0.984–0.989	Excellent
**all progesterone levels**	gen 2 vs Architect	121	0.814 95%CI: 0.743–0.866	Excellent	0.897 95%CI: 0.853–0.928	Excellent
**all progesterone levels**	gen 3 vs Architect	121	0.957 95%CI: 0.939–0.970	Excellent	0.978 95%CI: 0.969–0.985	Excellent
**Progesterone levels ≥ 1.5 ng/ml**	gen 2 vs gen 3	34	0.966 95%CI: 0.933–0.983	Excellent	0.983 95%CI: 0.9965–0.991	Excellent
**Progesterone levels ≥ 1.5 ng/ml**	gen 2 vs Architect	14	0.287 95%CI: 0–0.698	Poor	0.446 95%CI: 0–0.822	Fair
**Progesterone levels ≥ 1.5 ng/ml**	gen 3 vs Architect	14	0.938 95%CI: 0.820–0.980	Excellent	0.968 95%CI: 0.901–0.990	Excellent
**Progesterone levels 1.0- < 1.5 ng/ml**	gen 2 vs gen 3	45	0.288 95%CI: 0–0.542	Poor	0.488 95%CI: 0–0.703	Fair
**Progesterone levels 1.0- < 1.5 ng/ml**	gen 2 vs Architect	26	0.315 95%CI: 0–0.621	Poor	0.479 95%CI: 0–0.766	Fair
**Progesterone levels 1.0- < 1.5 ng/ml**	gen 3 vs Architect	26	0.887 95%CI: 0.764–0.948	Excellent	0.940 95%CI: 0.866–0.973	Excellent
**Progesterone levels 0.8- < 1.0 ng/ml**	gen 2 vs gen 3	30	0.138 95%CI: 0–0.470	Poor	0.242 95%CI: 0–0.639	Poor
**Progesterone levels 0.8- < 1.0 ng/ml**	gen 2 vs Architect	9	0.127 95%CI: 0–0.702	Poor	0.225 95%CI: 0–0.825	Poor
**Progesterone levels 0.8- < 1.0 ng/ml**	gen 3 vs Architect	9	0.779 95%CI: 0.289–0.945	Excellent	0.876 95%CI: 0.449–0.972	Excellent
**Progesterone levels < 0.8 ng/ml**	gen 2 vs gen 3	304	0.544 95%CI: 0.459–0.618	Fair	0.704 95%CI: 0.629–0.764	Good
**Progesterone levels < 0.8 ng/ml**	gen 2 vs Architect	70	0.359 95%CI: 0.137–0.547	Poor	0.529 95%CI: 0.241–0.707	Fair
**Progesterone levels < 0.8 ng/ml**	gen 3 vs Architect	70	0.846 95%CI: 0.763–0.901	Excellent	0.917 95%CI: 0.866–0.948	Excellent
**Progesterone levels on trigger day**	gen 2 vs gen 3	72	0.851 95%CI: 0.771–0.904	Excellent	0.919 95%CI: 0.871–0.949	Excellent
**Progesterone levels on trigger day**	gen 2 vs Architect	72	0.803 95%CI: 0.702–0.872	Excellent	0.890 95%CI: 0.825–0.931	Excellent
**Progesterone levels on trigger day**	gen 3 vs Architect	72	0.955 95%CI: 0.929–0.971	Excellent	0.977 95%CI: 0.963–0.986	Excellent

Progesterone elevation during the late follicular phase in stimulated IVF cycles is a frequent event, which cannot be prevented by the administration of GnRH analogues and occurs with an incidence up to 38% of all stimulated cycles, independent from the protocol used for stimulation [10,20]. Premature progesterone elevation will not only lead to an endometrial advancement and therefore to an asynchrony between the endometrium and the implanting embryo but also to an impaired embryo quality resulting in reduced pregnancy rates and implantation failure in ART treatment [2]. Therefore, the reliability of serum progesterone measurements is of the utmost importance as the results form the basis on which clinical decisions may be made. Many studies have focused solely on the progesterone level on the day of final oocyte maturation when making the decision to proceed with a fresh embryo transfer (ET) or to electively cryopreserve all available embryos, postponing embryo transfer.

Monitoring of progesterone levels during the earlier phases of ovarian stimulation alerts the clinician to rising progesterone levels in a more timely manner when countermeasures can be instigated to avoid further progesterone elevations.

Due to new technologies in laboratory equipment and therefore subsequent changes in assays used, reproducibility of hormonal measurement results is extremely important for the clinician in order to take the appropriate decision. To evaluate reproducibility between different progesterone assays, we choose to compare the test results using the ICC, which describes how strongly units in the same group resemble each other. This comparison shows that the reproducibility of the results ranged from poor to excellent, depending on whether blood was taken on the day of final oocyte maturation or during ovarian stimulation and also depended on the progesterone range.

Meanwhile, several studies confirmed the negative impact of progesterone levels above 1.5 ng/ml on the outcome of ART treatments when despite the elevated progesterone levels fresh embryo transfers were performed. Unfortunately, data on the performance and precision of fully automated direct immunoassay platforms, especially in the lower range of detectable P concentrations (<2.5 ng/mL), are limited. For ART outcome, the sensitive progesterone level starts from 0.8 ng/ml as already from this level onwards a negative impact on the pregnancy rate was shown [11]. Patton et al. [14] performed a study to determine consistency of measured progesterone levels among four automatic immunoassay analyzers and their concordance with levels detected by liquid chromatography-tandem mass spectrometry (LC-MS/MS), focusing in particular on progesterone levels from 0.9–2.5 ng/ml. Their results suggested that the automated analyzers performed reasonably well across low concentrations of progesterone, however, progesterone levels as determined by LC-MS/MS were at times significantly different from P levels in three of the four analyzers.

The current analysis confirmed partially the findings of Patton et al. [14], that is we observed significant differences in the reproducibility of progesterone results, measured by different assays, when the results were stratified according to the progesterone ranges. Stratification into three different ranges of progesterone levels was performed (≤ 0.8 ng/ml; 0.8 ng/ml—< 1.0 ng/ml; 1.0 ng/ml—< 1.5 ng/ml), as reproducibility of progesterone measurements in the lower ranges is especially critical in order to detect and prevent an early premature progesterone rise [21]. Whereas the reliability was excellent between the three assays when all progesterone levels were analysed and for the results obtained from the day of final oocyte maturation, poor reliability was found between „gen II” and „ gen III” when the progesterone levels were divided into different progesterone ranges. However, and this is a limitation of this study, wide confidence intervals due to small sample sizes may reduce the meaningfulness of those data and therefore future studies, including larger sample sizes should be conducted. Further on, this analysis was limited to the comparison of three progesterone assays while there is a wide range of assays on the market and our results can´t be generalized towards other progesterone assays.

Despite those limitations the herein reported findings underline the importance of a critical approach of the clinician towards the use and the comparability of progesterone results, obtained by different assays. This approach is especially important in lower progesterone ranges, as the detection of early progesterone rise will enable the clinician to adjust the ovarian stimulation regimen, e.g. reduction of the stimulation dosage [22] or shorten the stimulation duration [23], as these are strategies proven to prevent a possible progesterone elevation [21].

## Conclusions

Due to the complexity of IVF protocols and the still limited success rates, strategies should be adopted to maximise the chance of a positive outcome. Part of this process is to individualize ovarian stimulation with dose adaptation, based on the individual response of the patient and follicular dynamics. For this individualization, reliable assessment through evaluation of the endocrine profile is imperative. The current findings indicate that the reproducibility of progesterone levels, measured with different assays, are limited, especially in progesterone ranges below 1.5 ng/ml. In the herein presented comparison of the progesterone assays “gen II” versus “gen III” versus “Architect”, an excellent reproducibility of progesterone results throughout all ranges of progesterone levels was seen between the assays “gen III” and “Architect”, however varying reproducibility between the other assays.

Therefore, as a clinical consequence of these findings, progesterone thresholds, based on specific immunoassay platforms cannot be applied to progesterone results, obtained from other assays and it has to be kept in mind that this lack of reproducibility may lead to substantially different treatment decisions in ART-treatment. Consequently, also the reliability of progesterone thresholds, provided from previously published meta-analysis on that topic has to be questioned, since different assays were used in the studies included in that meta-analysis.

## Supporting information

S1 Table Reproducibility of the progesterone assays „gen 2“, „gen 3”and „Architect”according to Intraclass Correlation Coefficient (ICC) and interpretation according to Cicchetti et al. [19], with and without (highlighted in grey) inclusion of progesterone results below assay detection range. (DOCX)

S1 File Product information ROCHE Cobas Progesterone II. (PDF)

S2 File Product information ROCHE Cobas Progesterone III. (PDF)

S3 File Product information ABBOTT Architect System Progesterone. (PDF)

S4 File Progesterone results. (XLSX)
