# Supplementary material for: Inter-assay variation and reproducibility of progesterone measurements during ovarian stimulation for IVF
Source: PLoS One. 2018 Nov 1;13(11):e0206098. doi: 10.1371/journal.pone.0206098 (PMC6211677; doi:10.1371/journal.pone.0206098)
Supplement: S1 Table — (DOCX) [file pone.0206098.s001.docx]

**S1_Table: Reproducibility of the progesterone assays „gen 2“, „gen 3“ and „Architect“ according to Intraclass Correlation Coefficient (ICC) and interpretation according to Cicchetti et al.** [19**], with and without (highlighted in grey) inclusion of progesterone results below assay detection range.**

|  | **Comparison between** | **No of cases** | **ICC single measures** | **Interpretation** | **ICC average measures** | **Interpretation** |
| --- | --- | --- | --- | --- | --- | --- |
| **all progesterone levels** | gen 2 vs gen 3 | 413  341 | 0.973  95%CI: 0.968 – 0.979  0.973  95% CI: 0.967 – 0.978 | Excellent  Excellent | 0.986  95%CI: 0.984 – 0.989  0.986  95%CI 0.983 – 0.989 | Excellent  Excellent |
| **all progesterone levels** | gen 2 vs Architect | 121  120 | 0.814  95%CI: 0.743 – 0.866  0.810  95%CI: 0.738 – 0.863 | Excellent  Excellent | 0.897  95%CI: 0.853 – 0.928  0.895  95%CI: 0.849 – 0.927 | Excellent  Excellent |
| **all progesterone levels** | gen 3 vs Architect | 121  109 | 0.957  95%CI: 0.939 – 0.970  0.955  95%CI: 0.935 – 0.969 | Excellent  Excellent | 0.978  95%CI: 0.969 – 0.985  0.977  95%CI: 0.966 – 0.984 | Excellent  Excellent |
|  |  |  |  |  |  |  |
| **Progesterone levels ≥ 1.5 ng/ml** | gen 2 vs gen 3 | 34 | 0.966  95%CI: 0.933 – 0.983 | Excellent | 0.983  95%CI: 0.9965 – 0.991 | Excellent |
| **Progesterone levels ≥ 1.5 ng/ml** | gen 2 vs Architect | 14 | 0.287  95%CI: 0 – 0.698 | Poor | 0.446  95%CI: 0 – 0.822 | Fair |
| **Progesterone levels ≥ 1.5 ng/ml** | gen 3 vs Architect | 14 | 0.938  95%CI: 0.820 – 0.980 | Excellent | 0.968  95%CI: 0.901 – 0.990 | Excellent |
|  |  |  |  |  |  |  |
| **Progesterone levels 1.0- < 1.5 ng/ml** | gen 2 vs gen 3 | 45 | 0.288  95%CI: 0 – 0.542 | Poor | 0.488  95%CI: 0 – 0.703 | Fair |
| **Progesterone levels 1.0- < 1.5 ng/ml** | gen 2 vs Architect | 26 | 0.315  95%CI: 0 – 0.621 | Poor | 0.479  95%CI: 0 – 0.766 | Fair |
| **Progesterone levels 1.0- < 1.5 ng/ml** | gen 3 vs Architect | 26 | 0.887  95%CI: 0.764 – 0.948 | Excellent | 0.940  95%CI: 0.866 – 0.973 | Excellent |
|  |  |  |  |  |  |  |
| **Progesterone levels 0.8- < 1.0 ng/ml** | gen 2 vs gen 3 | 30 | 0.138  95%CI: 0 – 0.470 | Poor | 0.242  95%CI: 0 – 0.639 | Poor |
| **Progesterone levels 0.8- < 1.0 ng/ml** | gen 2 vs Architect | 9 | 0.127  95%CI: 0 – 0.702 | Poor | 0.225  95%CI: 0 – 0.825 | Poor |
| **Progesterone levels 0.8- < 1.0 ng/ml** | gen 3 vs Architect | 9 | 0.779  95%CI: 0.289 – 0.945 | Excellent | 0.876  95%CI: 0.449 – 0.972 | Excellent |
|  |  |  |  |  |  |  |
| **Progesterone levels < 0.8 ng/ml** | gen 2 vs gen 3 | 304  232 | 0.544  95%CI: 0.459 – 0.618  0.451  95%CI: 0.342 – 0.548 | Fair  Fair | 0.704  95%CI: 0.629 – 0.764  0.622  95%CI: 0.510 – 0.708 | Good  Good |
| **Progesterone levels < 0.8 ng/ml** | gen 2 vs Architect | 70  69 | 0.359  95%CI: 0.137 – 0.547  0.316  95%CI: 0.087 – 0.513 | Poor  Poor | 0.529  95%CI: 0.241 – 0.707  0.480  95%CI: 0.160 – 0.678 | Fair  Fair |
| **Progesterone levels < 0.8 ng/ml** | gen 3 vs Architect | 70  58 | 0.846  95%CI: 0.763 – 0.901  0.827  95%CI: 0.724 – 0.894 | Excellent  Excellent | 0.917  95%CI: 0.866 – 0.948  0.905  95%CI: 0.840 – 0.944 | Excellent  Excellent |
|  |  |  |  |  |  |  |
| **Progesterone levels on trigger day** | gen 2 vs gen 3 | 72 | 0.851  95%CI: 0.771 – 0.904 | Excellent | 0.919  95%CI: 0.871 – 0.949 | Excellent |
| **Progesterone levels on trigger day** | gen 2 vs Architect | 72 | 0.803  95%CI: 0.702 – 0.872 | Excellent | 0.890  95%CI: 0.825 -0.931 | Excellent |
| **Progesterone levels on trigger day** | gen 3 vs Architect | 72 | 0.955  95%CI: 0.929 – 0.971 | Excellent | 0.977  95%CI: 0.963 – 0.986 | Excellent |
