# Supplementary material for: Inter-assay variation and reproducibility of progesterone measurements during ovarian stimulation for IVF
Source: PLoS One. 2018 Nov 1;13(11):e0206098. doi: 10.1371/journal.pone.0206098 (PMC6211677; doi:10.1371/journal.pone.0206098)
Supplement: S1 File — (PDF) [file pone.0206098.s002.pdf]

Progesterone II

Progesterone

REF	Σ	SYSTEM
**12145383** 122	100	Elecsys 2010<br>MODULAR ANALYTICS E170<br>**cobas e** 411<br>**cobas e** 601<br>**cobas e** 602

English

Intended use

Immunoassay for the in vitro quantitative determination of progesterone in human serum and plasma.

The **e**lectro**c**hemi**l**uminescence **i**mmuno**a**ssay "ECLIA" is intended for use on Elecsys and **cobas e** immunoassay analyzers.

Summary

The gestagen progesterone is a steroid hormone having a molecular-weight of 314.5 daltons. Progesterone is mainly formed in the cells of the corpus luteum and during pregnancy in the placenta.

The progesterone concentration correlates with the development and regression of the corpus luteum. Whereas progesterone is barely detectable in the follicular phase of the female cycle, a rise in the progesterone level is observed one day prior to ovulation. Increased progesterone synthesis occurs during the luteal phase. In the second half of the cycle pregnanediol is excreted in urine as the main degradation product of progesterone.[1]

Progesterone brings about the conversion of the uterine mucosa into a tissue rich in glands (secretion phase), in order to prepare for the intrauterine implantation of the fertilized ovum. During pregnancy, progesterone inhibits the contraction of the myometrium. In the mammary gland, progesterone (together with estrogens) promotes the proliferation and secretion disposition of the alveoli.[1,2]

The determination of progesterone is utilized in fertility diagnosis for the detection of ovulation and assessment of the luteal phase.[2,3]

Test principle

Competition principle. Total duration of assay: 18 minutes.

- 1st incubation: 30 µL sample - in the presence of a biotinylated monoclonal progesterone-specific antibody and a progesterone derivative labeled with ruthenium complex[a)] - are incubated with Danazol to release progesterone. Progesterone from the sample competes with the labeled progesterone derivative for the antibody binding site.
- 2nd incubation: After addition of streptavidin-coated microparticles, the complex becomes bound to the solid phase via interaction of biotin and streptavidin. The amount of the labeled progesterone derivative bound to the solid phase is inversely proportional to the progesterone content of the sample.
- The reaction mixture is aspirated into the measuring cell where the microparticles are magnetically captured onto the surface of the electrode. Unbound substances are then removed with ProCell/ProCell M. Application of a voltage to the electrode then induces chemiluminescent emission which is measured by a photomultiplier.
- Results are determined via a calibration curve which is instrument-specifically generated by 2-point calibration and a master curve provided via the reagent barcode.

a) Tris(2,2'-bipyridyl)ruthenium(II)-complex ($Ru(bpy)_3^{2+}$)

Reagents - working solutions

The reagent rackpack is labeled as PROG II.

M Streptavidin-coated microparticles (transparent cap), 1 bottle, 6.5 mL:
Streptavidin-coated microparticles 0.72 mg/mL; preservative.

R1 Anti-progesterone-Ab~biotin (gray cap), 1 bottle, 10 mL:
Biotinylated monoclonal anti-progesterone antibody (mouse) 0.15 mg/L, phosphate buffer 25 mmol/L, pH 7.0; preservative.

R2 Progesterone-peptide~$Ru(bpy)_3^{2+}$ (black cap), 1 bottle, 8 mL:
Progesterone (of vegetable origin) coupled to a synthetic peptide labeled with ruthenium complex, 10 ng/mL; phosphate buffer 25 mmol/L, pH 7.0; preservative.

Precautions and warnings

For in vitro diagnostic use.
Exercise the normal precautions required for handling all laboratory reagents.
Disposal of all waste material should be in accordance with local guidelines.
Safety data sheet available for professional user on request.

Avoid foam formation in all reagents and sample types (specimens, calibrators and controls).

Reagent handling

The reagents in the kit have been assembled into a ready-for-use unit that cannot be separated.

All information required for correct operation is read in from the respective reagent barcodes.

Storage and stability

Store at 2-8 °C.

Do not freeze.

Store the Elecsys reagent kit **upright** in order to ensure complete availability of the microparticles during automatic mixing prior to use.

Stability:	
unopened at 2-8 °C	up to the stated expiration date
after opening at 2-8 °C	12 weeks
on the analyzers	8 weeks

Specimen collection and preparation

Only the specimens listed below were tested and found acceptable.

Serum collected using standard sampling tubes or tubes containing separating gel.

Na-, Li-heparin, K_3-EDTA, sodium citrate and sodium fluoride/potassium oxalate plasma. When sodium citrate is used, the results must be corrected by + 10 %.

Criterion: Recovery within 90-110 % of serum value or slope 0.9-1.1 + intercept within < ± 2x analytical sensitivity (LDL) + coefficient of correlation > 0.95.

Stable for 5 days at 2-8 °C, 6 months at -20 °C. Freeze only once.[4]

Stability of serum obtained with separating tubes: 48 hours at 2-8 °C (note the data provided by the tube manufacturer).

The sample types listed were tested with a selection of sample collection tubes that were commercially available at the time of testing, i.e. not all available tubes of all manufacturers were tested. Sample collection systems from various manufacturers may contain differing materials which could affect the test results in some cases. When processing samples in primary tubes (sample collection systems), follow the instructions of the tube manufacturer.

Centrifuge samples containing precipitates before performing the assay.

Do not use heat-inactivated samples.

Do not use samples and controls stabilized with azide.

Ensure the samples, calibrators and controls are at 20-25 °C prior to measurement.

Due to possible evaporation effects, samples, calibrators and controls on the analyzers should be analyzed/measured within 2 hours.

Progesterone II

Progesterone

Materials provided

See “Reagents – working solutions” section for reagents.

Materials required (but not provided)

- REF 12145391122, Progesterone II CalSet, for 4 x 1 mL
- REF 11731416190, PreciControl Universal, for 2 x 3 mL each of PreciControl Universal 1 and 2
- REF 03028542122, Diluent Estradiol/Progesterone, 2 x 22 mL sample diluent
- General laboratory equipment
- Elecsys 2010, MODULAR ANALYTICS E170 or **cobas e** analyzer

Accessories for Elecsys 2010 and **cobas e** 411 analyzers:

- REF 11662988122, ProCell, 6 x 380 mL system buffer
- REF 11662970122, CleanCell, 6 x 380 mL measuring cell cleaning solution
- REF 11930346122, Elecsys SysWash, 1 x 500 mL washwater additive
- REF 11933159001, Adapter for SysClean
- REF 11706802001, Elecsys 2010 AssayCup, 60 x 60 reaction vessels
- REF 11706799001, Elecsys 2010 AssayTip, 30 x 120 pipette tips

Accessories for MODULAR ANALYTICS E170, **cobas e** 601 and **cobas e** 602 analyzers:

- REF 04880340190, ProCell M, 2 x 2 L system buffer
- REF 04880293190, CleanCell M, 2 x 2 L measuring cell cleaning solution
- REF 03023141001, PC/CC-Cups, 12 cups to prewarm ProCell M and CleanCell M before use
- REF 03005712190, ProbeWash M, 12 x 70 mL cleaning solution for run finalization and rinsing during reagent change
- REF 12102137001, AssayTip/AssayCup Combimagazine M, 48 magazines x 84 reaction vessels or pipette tips, waste bags
- REF 03023150001, WasteLiner, waste bags
- REF 03027651001, SysClean Adapter M

Accessories for all analyzers:

- REF 11298500316, Elecsys SysClean, 5 x 100 mL system cleaning solution

Assay

For optimum performance of the assay follow the directions given in this document for the analyzer concerned. Refer to the appropriate operator’s manual for analyzer-specific assay instructions.

Resuspension of the microparticles takes place automatically prior to use. Read in the test-specific parameters via the reagent barcode. If in exceptional cases the barcode cannot be read, enter the 15-digit sequence of numbers.

Bring the cooled reagents to approx. 20 °C and place on the reagent disk (20 °C) of the analyzer. Avoid foam formation. The system automatically regulates the temperature of the reagents and the opening/closing of the bottles.

Calibration

Traceability: This method has been standardized via ID-GC/MS (“Isotope Dilution Gas Chromatography Mass Spectrometry”).[5]

Every Elecsys reagent set has a barcoded label containing specific information for calibration of the particular reagent lot. The predefined master curve is adapted to the analyzer using the relevant CalSet.

Calibration frequency: Calibration must be performed once per reagent lot using fresh reagent (i.e. not more than 24 hours since the reagent kit was registered on the analyzer). Renewed calibration is recommended as follows:

- after 1 month (28 days) when using the same reagent lot
- after 7 days (when using the same reagent kit on the analyzer)
- as required: e.g. quality control findings outside the defined limits

Quality control

For quality control, use PreciControl Universal.

In addition, other suitable control material can be used.

Controls for the various concentration ranges should be run individually at least once every 24 hours when the test is in use, once per reagent kit, and following each calibration.

The control intervals and limits should be adapted to each laboratory’s individual requirements. Values obtained should fall within the defined limits. Each laboratory should establish corrective measures to be taken if values fall outside the defined limits.

Follow the applicable government regulations and local guidelines for quality control.

Calculation

The analyzer automatically calculates the analyte concentration of each sample (either in nmol/L, ng/mL or in µg/L).

Conversion factors:	nmol/L x 0.314 = ng/mL (µg/L)
	ng/mL x 3.18 = nmol/L

Limitations - interference

The assay is unaffected by icterus (bilirubin ditaurate < 923 µmol/L or < 54 mg/dL), hemolysis (Hb < 0.621 mmol/L or < 1.0 g/dL), lipemia (Intralipid < 720 mg/dL) and biotin (< 82 nmol/L or < 20 ng/mL).

Criterion: Recovery within ± 10 % of initial value.

Samples should not be taken from patients receiving therapy with high biotin doses (i.e. > 5 mg/day) until at least 8 hours following the last biotin administration.

No interference was observed from rheumatoid factors up to a concentration of 2000 IU/mL.

In vitro tests were performed on 18 commonly used pharmaceuticals. Of these, only phenylbutazone at therapeutic dosage levels showed interference with the assay (progesterone values depressed).

In rare cases, interference due to extremely high titers of antibodies to analyte-specific antibodies, streptavidin or ruthenium can occur. These effects are minimized by suitable test design.

For diagnostic purposes, the results should always be assessed in conjunction with the patient’s medical history, clinical examination and other findings.

Limits and ranges

Measuring range

0.095-191 nmol/L or 0.030-60.0 ng/mL (defined by the lower detection limit and the maximum of the master curve). Values below the lower detection limit are reported as < 0.095 nmol/L or < 0.030 ng/mL. Values above the measuring range are reported as > 191 nmol/L or > 60.0 ng/mL.

Lower limits of measurement

Lower detection limit of the test

Lower detection limit: 0.095 nmol/L (0.030 ng/mL)

The lower detection limit represents the lowest measurable analyte level that can be distinguished from zero. It is calculated as the value lying two standard deviations above that of the lowest standard (master calibrator, standard 1 + 2 SD, repeatability study, n = 21).

Dilution

Samples with progesterone concentrations above the measuring range can be diluted with Diluent Estradiol/Progesterone or a suitable human serum with a low analyte concentration. The recommended dilution is 1:10. The concentration of the diluted sample must be > 6 nmol/L (> 2 ng/mL).

After dilution, multiply the result by the dilution factor.

Depending on the biological variance of the diluted patient sample and the human serum matrix used for production of Diluent Estradiol/Progesterone, lower recovery of diluted samples may be observed.

Expected values

Studies with the Elecsys Progesterone II assay have revealed the following progesterone values:

Progesterone II

Progesterone

Test subjects	N	Percentiles			
		50th	5-95th	50th	5-95th
		nmol/L		ng/mL	
Men	33	1.8	0.7-4.3	0.6	0.2-1.4
Women					
• Follicular phase	192	2.1	0.6-4.7	0.7	0.2-1.5
• Ovulation phase	13	3.9	2.4-9.4	1.2	0.8-3.0
• Luteal phase	158	36	5.3-86	11	1.7-27
• Postmenopause	89	1.0	0.3-2.5	0.3	0.1-0.8

Elecsys Progesterone II Multicenter Evaluation, 5/99

Each laboratory should investigate the transferability of the expected values to its own patient population and if necessary determine its own reference ranges.

Specific performance data

Representative performance data on the analyzers are given below. Results obtained in individual laboratories may differ.

Precision

Precision was determined using Elecsys reagents, pooled human sera and controls in a modified protocol (EP5-A) of the CLSI (Clinical and Laboratory Standards Institute): 6 times daily for 10 days (n = 60); repeatability on MODULAR ANALYTICS E170 analyzer, n = 21. The following results were obtained:

Elecsys 2010 and **cobas e** 411 analyzers					
			**Repeatability**		
Sample	Mean		SD		CV
	nmol/L	ng/mL	nmol/L	ng/mL	%
Human serum 1	4.99	1.57	0.13	0.04	2.4
Human serum 2	38.2	12.0	0.54	0.17	1.5
Human serum 3	96.0	30.2	2.42	0.76	2.7
PreciControl U[b)]1	28.1	8.83	0.60	0.19	2.3
PreciControl U2	66.1	20.8	1.11	0.35	1.7

b) U = Universal

Elecsys 2010 and **cobas e** 411 analyzers					
			**Intermediate precision**		
Sample	Mean		SD		CV
	nmol/L	ng/mL	nmol/L	ng/mL	%
Human serum 1	4.99	1.57	0.29	0.09	5.4
Human serum 2	38.2	12.0	1.53	0.48	4.1
Human serum 3	96.0	30.2	4.90	1.54	5.5
PreciControl U1	28.1	8.83	1.21	0.38	4.6
PreciControl U2	66.1	20.8	2.45	0.77	3.7

MODULAR ANALYTICS E170, **cobas e** 601 and **cobas e** 602 analyzers					
	**Repeatability**				
Sample	Mean		SD		CV
	nmol/L	ng/mL	nmol/L	ng/mL	%
Human serum 1	2.31	0.73	0.07	0.02	2.9
Human serum 2	9.57	3.01	0.13	0.04	1.4
Human serum 3	103	32.4	0.96	0.30	0.9
PreciControl U1	15.6	4.89	0.17	0.05	1.1
PreciControl U2	61.5	19.3	0.45	0.14	0.7

MODULAR ANALYTICS E170, **cobas e** 601 and **cobas e** 602 analyzers					
	**Intermediate precision**				
Sample	Mean		SD		CV
	nmol/L	ng/mL	nmol/L	ng/mL	%
Human serum 1	2.52	0.79	0.12	0.04	4.8
Human serum 2	10.0	3.13	0.28	0.09	2.8
Human serum 3	112	35.3	2.23	0.70	2.0
PreciControl U1	16.7	5.26	0.55	0.17	3.3
PreciControl U2	64.7	20.4	1.19	0.38	1.8

Method comparison

A comparison of the Elecsys Progesterone II assay (y) with the Elecsys Progesterone assay (x) using clinical samples gave the following correlations (ng/mL):

Number of samples measured: 88

Passing/Bablok[6]	Linear regression
y = 0.91x + 0.29	y = 0.90x + 0.50
τ = 0.933	r = 0.998

The sample concentrations were between approximately 0.6 and 140 nmol/L (approximately 0.2 and 44 ng/mL).

Analytical specificity

For the antibody derivative used, the following cross-reactivities were found (in %):

Androstendiol	0.002
Androstendione	0.136
Corticosterone	0.687
Cortisol	0.005
Danazol	0.002
DHEA-S	0.009
D-(-)-Norgestrel	0.008
Estradiol	0.009
Ethisterone	0.002
Ethinoldioldiacetate	n.d.[c)]
Medroxyprogesterone	0.812
Norethindrone	0.010
Norethindrone acetate	n.d.
Testosterone	0.020
4-Pregnen-11β-17α-diol-3,20-dione	0.338
11-Deoxycorticosterone	0.296
11-Deoxycortisol	0.392
5-α-Dihydrotestosterone	0.040
5-β-Dihydroprogesterone	20.7
5α-Pregnen-3β-ol-20-one	0.858
5β-Pregnan-3α-ol-20-one	0.211
6α-Methyl-17α-hydroxy-progesterone acetate	0.257
6α-Methylprednisolone	n.d.
17α-Hydroxypregnenolone	0.018
17α-Hydroxyprogesterone	1.30
20α-Hydroxy-4-pregnen-3-one	0.016

c) n.d. = not detectable

Functional sensitivity

0.48 nmol/L (0.15 ng/mL)

Progesterone II

Progesterone

cobas®

The functional sensitivity is the lowest analyte concentration that can be reproducibly measured with an intermediate precision CV of < 20 %.

References

1 Johnson MR, Carter G, Grint C, et al. Relationship between ovarian steroids, gonadotrophins and relaxin during the menstrual cycle. Acta Endocrinol 1993;129:121-125.

2 Runnebaum B, Rabe T. Gynäkologische Endokrinologie und Fortpflanzungsmedizin Springer Verlag 1994; Band 1:36-38,70,116 Band 2:137,360,398-399,408-409,422-423. ISBN 3-540-57345-3, ISBN 3-540-57347-x.

3 Guillaume J, Benjamin F, Sicuranza B, et al. Maternal serum levels of estradiol, progesterone and h-Choriongonadotropin in ectopic pregnancy and their correlation with endometrial histologic findings. Surg Gynecol Obstet 1987;165:9-12.

4 Wu AHB. Tietz Clinical Guide To Laboratry Tests, 4th Edition, WB Saunders Co, 2006:894 pp.

5 Thienpont LM, Verhseghe PG, Van Brussel KA, et al. Efforts by industry toward standardization of serum estradiol-17β measurements. Clin Chem 1998;44(3):671-674.

6 Bablok W, Passing H, Bender R, et al. A general regression procedure for method transformation. Application of linear regression procedures for method comparison studies in clinical chemistry, Part III. J Clin Chem Clin Biochem 1988 Nov;26(11):783-790.

For further information, please refer to the appropriate operator's manual for the analyzer concerned, the respective application sheets, the product information and the Method Sheets of all necessary components (if available in your country).

A point (period/stop) is always used in this Method Sheet as the decimal separator to mark the border between the integral and the fractional parts of a decimal numeral. Separators for thousands are not used.

Symbols

Roche Diagnostics uses the following symbols and signs in addition to those listed in the ISO 15223-1 standard.

CONTENT	Contents of kit
SYSTEM	Analyzers/Instruments on which reagents can be used
REAGENT	Reagent
CALIBRATOR	Calibrator
⟶	Volume after reconstitution or mixing

COBAS, COBAS E, ELECSYS, MODULAR and PRECICONTROL are trademarks of Roche. INTRALIPID is a trademark of Fresenius Kabi AB.

All other product names and trademarks are the property of their respective owners.

Significant additions or changes are indicated by a change bar in the margin.

© 2013, Roche Diagnostics

CE

Roche Diagnostics GmbH, Sandhofer Strasse 116, D-68305 Mannheim
www.roche.com

Roche
