# Supplementary material for: Inter-assay variation and reproducibility of progesterone measurements during ovarian stimulation for IVF
Source: PLoS One. 2018 Nov 1;13(11):e0206098. doi: 10.1371/journal.pone.0206098 (PMC6211677; doi:10.1371/journal.pone.0206098)
Supplement: S2 File — (PDF) [file pone.0206098.s003.pdf]

07027699500V2.0

Elecsys Progesterone III

cobas®

REF	Instructions for use	Σ	SYSTEM
07027699190	07027699500	300	**cobas e** 801

English

System Information

Short name	ACN (application code number)
PROG 3	10045

Intended use

Immunoassay for the in vitro quantitative determination of progesterone in human serum and plasma.

The **e**lectro**c**hemi**l**uminescence **i**mmuno**a**ssay "ECLIA" is intended for use on the **cobas e** 801 immunoassay analyzer.

Summary

The gestagen progesterone is a steroid hormone which is mainly formed in the cells of the corpus luteum and during pregnancy in the placenta.

The progesterone concentration correlates with the development and regression of the corpus luteum. Whereas progesterone is barely detectable in the follicular phase of the female cycle, a rise in the progesterone level is observed one day prior to ovulation. Increased progesterone synthesis occurs during the luteal phase. In the second half of the cycle pregnanediol is excreted in urine as the main degradation product of progesterone.[1]

Progesterone brings about the conversion of the uterine mucosa into a tissue rich in glands (secretion phase), in order to prepare for the intrauterine implantation of the fertilized ovum. During pregnancy, progesterone inhibits the contraction of the myometrium. In the mammary gland, progesterone (together with estrogens) promotes the proliferation, secretion and disposition of the alveoli.[1,2,3,4]

The determination of progesterone is utilized in fertility diagnosis for the detection of ovulation and assessment of the luteal phase.[5]

Test principle

Competition principle. Total duration of assay: 18 minutes.

- 1st incubation: By incubating the sample (12 µL) with a progesterone-specific biotinylated antibody, immunocomplexes are formed, the amount of which is dependent upon the analyte concentration in the sample.
- 2nd incubation: After addition of streptavidin-coated microparticles and an progesterone derivative labeled with a ruthenium complex[a)], the still-vacant sites of the biotinylated antibodies become occupied, with formation of an antibody-hapten complex. The entire complex becomes bound to the solid phase via interaction of biotin and streptavidin.
- The reaction mixture is aspirated into the measuring cell where the microparticles are magnetically captured onto the surface of the electrode. Unbound substances are then removed with ProCell II M. Application of a voltage to the electrode then induces chemiluminescent emission which is measured by a photomultiplier.
- Results are determined via a calibration curve which is instrument-specifically generated by 2-point calibration and a master curve provided via the **cobas** link.

a) Tris(2,2'-bipyridyl)ruthenium(II)-complex ($Ru(bpy)_3^{2+}$)

Reagents - working solutions

The **cobas e** pack is labeled as PROG 3.

M Streptavidin-coated microparticles, 1 bottle, 12.4 mL:
Streptavidin-coated microparticles 0.72 mg/mL; preservative.

R1 Anti-progesterone-Ab~biotin, 1 bottle, 21.0 mL:
Biotinylated monoclonal anti-progesterone antibody (recombinant, sheep) 30 ng/mL, phosphate buffer 25 mmol/L, pH 7.0; preservative.

R2 Progesterone-peptide~$Ru(bpy)_3^{2+}$, 1 bottle, 18.8 mL:
Progesterone (of vegetable origin) coupled to a synthetic peptide labeled with ruthenium complex, 2 ng/mL; phosphate buffer 25 mmol/L, pH 7.0; preservative.

Precautions and warnings

For in vitro diagnostic use.
Exercise the normal precautions required for handling all laboratory reagents.
Disposal of all waste material should be in accordance with local guidelines.
Safety data sheet available for professional user on request.

Avoid foam formation in all reagents and sample types (specimens, calibrators and controls).

Reagent handling

The reagents in the kit have been assembled into a ready-for-use unit that cannot be separated.

All information required for correct operation is available via the **cobas** link.

Storage and stability

Store at 2-8 °C.

Do not freeze.

Store the **cobas e** pack **upright** in order to ensure complete availability of the microparticles during automatic mixing prior to use.

Stability:	
unopened at 2-8 °C	up to the stated expiration date
on the **cobas e** 801 analyzer	16 weeks

Specimen collection and preparation

Only the specimens listed below were tested and found acceptable.

Serum collected using standard sampling tubes or tubes containing separating gel.

Li-heparin, K_2-EDTA and K_3-EDTA plasma.

Criterion: Slope 0.9-1.1 + intercept within ≤ ± 0.1 ng/mL + coefficient of correlation ≥ 0.95.

Stable for 1 day at 20-25 °C, 5 days at 2-8 °C, 6 months at -20 °C (± 5 °C). Freeze only once.

The sample types listed were tested with a selection of sample collection tubes that were commercially available at the time of testing, i.e. not all available tubes of all manufacturers were tested. Sample collection systems from various manufacturers may contain differing materials which could affect the test results in some cases. When processing samples in primary tubes (sample collection systems), follow the instructions of the tube manufacturer.

Centrifuge samples containing precipitates before performing the assay.

Do not use heat-inactivated samples.

Do not use samples and controls stabilized with azide.

Ensure the samples and calibrators are at 20-25 °C prior to measurement.

Due to possible evaporation effects, samples and calibrators on the analyzers should be analyzed/measured within 2 hours.

Materials provided

See "Reagents – working solutions" section for reagents.

Materials required (but not provided)

- REF 07092547190, Progesterone III CalSet, for 4 x 1.0 mL
- REF 11731416190, PreciControl Universal, for 4 x 3 mL
- REF 03028542122, Diluent Estradiol/Progesterone, 2 x 22 mL sample diluent
- General laboratory equipment
- **cobas e** 801 analyzer

Accessories for the **cobas e** 801 analyzer:

- REF 06908799190, ProCell II M, 2 x 2 L system solution
- REF 04880293190, CleanCell M, 2 x 2 L measuring cell cleaning solution
- REF 07485409001, Reservoir Cups, 8 cups to supply ProCell II M and CleanCell M

Elecsys Progesterone III

cobas®

- REF 06908853190, PreClean II M, 2 x 2 L wash solution
- REF 05694302001, Assay Tip/Assay Cup tray, 6 magazines x 6 magazine stacks x 105 assay tips and 105 assay cups, 3 wasteliners
- REF 07485425001, Liquid Flow Cleaning Cup, 2 adaptor cups to supply ISE Cleaning Solution/Elecsys SysClean for Liquid Flow Cleaning Detection Unit
- REF 07485433001, PreWash Liquid Flow Cleaning Cup, 1 adaptor cup to supply ISE Cleaning Solution/Elecsys SysClean for Liquid Flow Cleaning PreWash Unit
- REF 11298500316, ISE Cleaning Solution/Elecsys SysClean, 5 x 100 mL system cleaning solution

Assay

For optimum performance of the assay follow the directions given in this document for the analyzer concerned. Refer to the appropriate operator's manual for analyzer-specific assay instructions.

Resuspension of the microparticles takes place automatically prior to use.

Place the cooled (stored at 2-8 °C) **cobas e** pack on the reagent manager. Avoid foam formation. The system automatically regulates the temperature of the reagents and the opening/closing of the **cobas e** pack.

Calibration

Traceability: The Elecsys Progesterone III assay is traceable via ID-GC/MS (isotope dilution gas chromatography/mass spectrometry) to highly purified progesterone by weight analogously to BCR-348R and ERM-DA347.[6]

The predefined master curve is adapted to the analyzer using the relevant CalSet.

Calibration frequency: Calibration must be performed once per reagent lot using fresh reagent (i.e. not more than 24 hours since the **cobas e** pack was registered on the analyzer).

Calibration interval may be extended based on acceptable verification of calibration by the laboratory.

Renewed calibration is recommended as follows:

- after 8 weeks when using the same reagent lot
- after 28 days when using the same **cobas e** pack on the analyzer
- as required: e.g. quality control findings outside the defined limits

Quality control

For quality control, use PreciControl Universal.

In addition, other suitable control material can be used.

Controls for the various concentration ranges should be run individually at least once every 24 hours when the test is in use, once per **cobas e** pack, and following each calibration.

The control intervals and limits should be adapted to each laboratory's individual requirements. Values obtained should fall within the defined limits. Each laboratory should establish corrective measures to be taken if values fall outside the defined limits.

If necessary, repeat the measurement of the samples concerned.

Follow the applicable government regulations and local guidelines for quality control.

Calculation

The analyzer automatically calculates the analyte concentration of each sample (either in nmol/L, ng/mL or in µg/L).

Conversion factors: nmol/L x 0.314 = ng/mL (µg/L)
ng/mL x 3.18 = nmol/L

Limitations - interference

The effect of the following endogenous substances and pharmaceutical compounds on assay performance was tested. Interferences were tested up to the listed concentrations and no impact on results was observed.

Endogenous substances

Compound	Concentration tested
Bilirubin	≤ 923 µmol/L or ≤ 54 mg/dL
Hemoglobin	≤ 0.621 mmol/L or ≤ 1000 mg/dL
Intralipid	≤ 200 mg/dL
Biotin	≤ 123 nmol/L or ≤ 30 ng/mL
Rheumatoid factors	≤ 1200 IU/mL
IgG	≤ 7 g/dL
IgA	≤ 0.4 g/dL
IgM	≤ 1 g/dL

Criterion: Recovery within ± 10 % of initial value for samples > 2 ng/mL, ± 15 % for samples > 0.5 to 2 ng/mL and ± 0.2 ng/mL for samples ≤ 0.5 ng/mL.

Visibly turbid samples give a false low result.

Samples should not be taken from patients receiving therapy with high biotin doses (i.e. > 5 mg/day) until at least 8 hours following the last biotin administration.

Pharmaceutical substances

In vitro tests were performed on 16 commonly used pharmaceuticals. Of these, only phenylbutazone at therapeutic dosage levels showed interference with the assay (progesterone values depressed).

In addition, the following special drug was tested. No interference with the assay was found.

Special drug

Drug	Concentration tested mg/L
Clomiphene citrate	100

In rare cases, interference due to extremely high titers of antibodies to analyte-specific antibodies, streptavidin or ruthenium can occur. These effects are minimized by suitable test design.

For diagnostic purposes, the results should always be assessed in conjunction with the patient's medical history, clinical examination and other findings.

Limits and ranges

Measuring range

0.159-191 nmol/L or 0.05-60 ng/mL (defined by the Limit of Detection and the maximum of the master curve). Values below the Limit of Detection are reported as < 0.159 nmol/L or < 0.05 ng/mL. Values above the measuring range are reported as > 191 nmol/L or > 60 ng/mL.

Lower limits of measurement

Limit of Blank, Limit of Detection and Limit of Quantitation

Limit of Blank = 0.080 nmol/L (0.025 ng/mL)

Limit of Detection = 0.159 nmol/L (0.05 ng/mL)

Limit of Quantitation = 0.636 nmol/L (0.2 ng/mL)

The Limit of Blank, Limit of Detection and Limit of Quantitation were determined in accordance with the CLSI (Clinical and Laboratory Standards Institute) EP17-A2 requirements.

The Limit of Blank is the 95th percentile value from n ≥ 60 measurements of analyte-free samples over several independent series. The Limit of Blank corresponds to the concentration below which analyte-free samples are found with a probability of 95 %.

The Limit of Detection is determined based on the Limit of Blank and the standard deviation of low concentration samples. The Limit of Detection corresponds to the lowest analyte concentration which can be detected (value above the Limit of Blank with a probability of 95 %).

The Limit of Quantitation is defined as the lowest amount of analyte in a sample that can be accurately quantitated with a total allowable relative error of ≤ 20 %.

Dilution

Samples with progesterone concentrations above the measuring range can be diluted with Diluent Estradiol/Progesterone or a suitable human serum with a low analyte concentration. The recommended dilution is 1:10. The concentration of the diluted sample must be ≥ 3.18 nmol/L (≥ 1 ng/mL).

After manual dilution, multiply the result by the dilution factor.

Elecsys Progesterone III

cobas®

Depending on the biological variance of the diluted patient sample and the human serum matrix used for production of Diluent Estradiol/Progesterone, lower recovery of diluted samples may be observed.

Expected values

The expected ranges were determined by testing specimens drawn from 147 apparently healthy males, 142 apparently healthy, post-menopausal women over the age of 50, and from 416 apparently healthy pregnant women between the ages of 17 and 45 (137 in the first trimester, 140 in the second trimester, and 139 in the third trimester). The expected range for healthy women was determined by weekly blood drawing over a period of 3 months from 26 apparently healthy women between the ages of 18 and 45 that were not taking any hormonal contraceptives. Based on a central 90 % interval, the following ranges were obtained:

Test subjects	N	5th percentile nmol/L	Median nmol/L	95th percentile nmol/L
Healthy men	147	< 0.159	< 0.159	0.474
Healthy women				
Follicular phase	117	0.181	0.588	2.84
Ovulation phase	38	0.385	1.60	38.1
Luteal phase	126	5.82	31.9	75.9
Postmenopause	142	< 0.159	< 0.159	0.401
Healthy pregnant women				
1st trimester	137	35.0	76.3	141
2nd trimester	140	80.8	151	264
3rd trimester	139	187	340	681

Test subjects	N	5th percentile ng/mL	Median ng/mL	95th percentile ng/mL
Healthy men	147	< 0.05	< 0.05	0.149
Healthy women				
Follicular phase	117	0.057	0.185	0.893
Ovulation phase	38	0.121	0.503	12.0
Luteal phase	126	1.83	10.0	23.9
Postmenopause	142	< 0.05	< 0.05	0.126
Healthy pregnant women				
1st trimester	137	11.0	24.0	44.3
2nd trimester	140	25.4	47.5	83.3
3rd trimester	139	58.7	107	214

Each laboratory should investigate the transferability of the expected values to its own patient population and if necessary determine its own reference ranges.

Specific performance data

Representative performance data on the analyzer is given below. Results obtained in individual laboratories may differ.

Precision

Precision was determined using Elecsys reagents, pooled human sera and controls in a protocol (EP05-A3) of the CLSI (Clinical and Laboratory Standards Institute): 2 runs per day in duplicate each for 21 days (n = 84). The following results were obtained:

**cobas e** 801 analyzer					
			**Repeatability**		
Sample	Mean		SD		CV
	nmol/L	ng/mL	nmol/L	ng/mL	%
Human serum 1	0.172	0.054	0.035	0.011	20.7
Human serum 2	2.10	0.659	0.089	0.028	4.2
Human serum 3	9.64	3.03	0.264	0.083	2.7
Human serum 4	70.0	22.0	0.789	0.248	1.1
Human serum 5	170	53.5	1.84	0.579	1.1
PreciControl U[b)]1	23.9	7.52	0.480	0.151	2.0
PreciControl U2	49.6	15.6	0.712	0.224	1.4

b) U = Universal

**cobas e** 801 analyzer					
			**Intermediate precision**		
Sample	Mean		SD		CV
	nmol/L	ng/mL	nmol/L	ng/mL	%
Human serum 1	0.172	0.054	0.076	0.024	43.9
Human serum 2	2.10	0.659	0.130	0.041	6.2
Human serum 3	9.64	3.03	0.321	0.101	3.3
Human serum 4	70.0	22.0	1.18	0.372	1.7
Human serum 5	170	53.5	2.86	0.898	1.7
PreciControl U1	23.9	7.52	0.677	0.213	2.8
PreciControl U2	49.6	15.6	0.989	0.311	2.0

Method comparison

A comparison of the Elecsys Progesterone III assay, REF 07027699190 (**cobas e** 801 analyzer; y) with the Elecsys Progesterone III assay, REF 07092539190 (**cobas e** 601 analyzer; x) gave the following correlations (ng/mL):

Number of samples measured: 153

Passing/Bablok[7]	Linear regression
y = 0.984x + 0.001	y = 0.981x + 0.086
τ = 0.985	r = 0.999

The sample concentrations were between 0.050 and 59.0 ng/mL.

Analytical specificity

For the Elecsys Progesterone III assay, the following cross-reactivities were found at the respective additive concentration, tested with progesterone concentrations of approximately 0.3 ng/mL and 5 ng/mL:

**Substance**	**Additive concentration ng/mL**	**Cross-reactivity %**
Androstenediol	4000	0.001
Androstenedione	80	0.107
Aldosterone	1000	0.003
Allopregnanolone	2000	0.347
Corticosterone	200	0.921
Cortisol	20000	0.006
Danazol	100000	0.001
DHEA-S	16000	n. d.[c)]
Norgestrel	1000	0.011
Estradiol	400	n. d.
Ethisterone	1000	0.001
Ethynodiol diacetate	1000	n. d.
Medroxyprogesterone	5000	0.004
Norethindrone	1000	0.004

Substance	Additive concentration ng/mL	Cross-reactivity %
Norethindrone acetate	1000	0.008
Testosterone	2000	0.069
21-Deoxycortisol	2000	0.067
11-Deoxycorticosterone	600	3.92
11-Deoxycortisol	6000	0.015
5-α-Dihydrotestosterone	20	n. d.
5-β-Dihydroprogesterone	240	0.366
Pregnenolone	16000	0.410
Pregnanolone	2000	0.145
Medroxyprogesterone acetate	1000	0.003
6α-Methylprednisolone	1000	0.003
17α-Hydroxypregnenolone	2000	0.009
17α-Hydroxyprogesterone	2000	0.066
20α-Hydroxy-4-pregnen-3-one	250	0.086

c) n. d. = not detectable

References

1 Johnson MR, Carter G, Grint C, et al. Relationship between ovarian steroids, gonadotrophins and relaxin during the menstrual cycle. Acta Endocrinol 1993;129:121-125.

2 Laufer N, Navot D, Schenker JG. The pattern of luteal phase plasma progesterone and estradiol in fertile cycles. Am J Obstet Gynecol 1982;143:808-813.

3 Veldhuis JD, Christiansen E, Evans WS, et al. Physiological profiles of episodic progesterone release during the midluteal phase of the human menstrual cycle: analysis of circadian and ultradian rhythms, discrete pulse properties, and correlations with simultaneous luteinizing hormone release. J Clin Endocrinol Metab 1988;66(2):414-421.

4 Filicori M, Butler JP, Crowley WF Jr. Neuroendocrine regulation of the corpus luteum in the human. J Clin Invest 1984;73:1638-1647.

5 Guillaume J, Benjamin F, Sicuranza B, et al. Maternal serum levels of estradiol, progesterone and h-Choriongonadotropin in ectopic pregnancy and their correlation with endometrial histologic findings Surg Gynecol Obstet 1987;165:9-12.

6 Thienpont L, Siekmann L, Lawson A, et al. Development, Validation and Certification by Isotope Dilution Gas Chromatography-Mass Spectrometry of Lyophilized Human Serum Reference Materials for Cortisol (CRM 192 and 193) and Progesterone (CRM 347 and 348). Clin Chem 1991;37(4):540-546.

7 Bablok W, Passing H, Bender R, et al. A general regression procedure for method transformation. Application of linear regression procedures for method comparison studies in clinical chemistry, Part III. J Clin Chem Clin Biochem 1988 Nov;26(11):783-790.

For further information, please refer to the appropriate operator’s manual for the analyzer concerned, the respective application sheets, the product information and the Method Sheets of all necessary components (if available in your country).

A point (period/stop) is always used in this Method Sheet as the decimal separator to mark the border between the integral and the fractional parts of a decimal numeral. Separators for thousands are not used.

Symbols

Roche Diagnostics uses the following symbols and signs in addition to those listed in the ISO 15223-1 standard (for USA: see https://usdiagnostics.roche.com for definition of symbols used):

CONTENT	Contents of kit
SYSTEM	Analyzers/Instruments on which reagents can be used
REAGENT	Reagent
CALIBRATOR	Calibrator
→	Volume after reconstitution or mixing
GTIN	Global Trade Item Number

COBAS, COBAS E, ELECSYS and PRECICONTROL are trademarks of Roche. INTRALIPID is a trademark of Fresenius Kabi AB.

All other product names and trademarks are the property of their respective owners.

Additions, deletions or changes are indicated by a change bar in the margin.

© 2017, Roche Diagnostics

Roche Diagnostics GmbH, Sandhofer Strasse 116, D-68305 Mannheim
www.roche.com
