# Supplementary material for: Inter-assay variation and reproducibility of progesterone measurements during ovarian stimulation for IVF
Source: PLoS One. 2018 Nov 1;13(11):e0206098. doi: 10.1371/journal.pone.0206098 (PMC6211677; doi:10.1371/journal.pone.0206098)
Supplement: S3 File — (PDF) [file pone.0206098.s004.pdf]

ARCHITECT
SYSTEM

en

Progesterone

REF 7K77

49-3265/R3

B7K770

Read Highlighted Changes
Revised April, 2010

Progesterone

Customer Service: Contact your local representative or find country specific contact information on www.abbottdiagnostics.com

Package insert instructions must be carefully followed. Reliability of assay results cannot be guaranteed if there are any deviations from the instructions in this package insert.

Key to symbols used

Symbol	Meaning	Symbol	Meaning
REF	List Number	CAL 1	Calibrator (1,2)
IVD	*In Vitro* Diagnostic Medical Device	CONTROL L	Control Low, Medium, High (L, M, H)
LOT	Lot Number	REAGENT LOT	Reagent Lot
(hourglass)	Expiration Date	REACTION VESSELS	Reaction Vessels
SN	Serial Number	SAMPLE CUPS	Sample Cups
2°C–8°C (thermometer)	Store at 2-8°C	SEPTUM	Septum
		REPLACEMENT CAPS	Replacement Caps
(book with i)	Consult instructions for use	WARNING: SENSITIZER	Warning: May cause an allergic reaction
(factory)	Manufacturer	CONTAINS: AZIDE	Contains sodium azide. Contact with acids liberates very toxic gas.

See **REAGENTS** section for a full explanation of symbols used in reagent component naming.

Abbott

NAME

ARCHITECT Progesterone

INTENDED USE

The ARCHITECT Progesterone assay is a Chemiluminescent Microparticle Immunoassay (CMIA) for the quantitative determination of progesterone in human serum and plasma.

SUMMARY AND EXPLANATION OF TEST

Progesterone is produced primarily by the corpus luteum of the ovary in normally menstruating women and to a lesser extent by the adrenal cortex.[1] At approximately the 6th week of pregnancy, the placenta becomes the major producer of progesterone.[2-5] The major functions of progesterone are in the preparation of the uterus for implantation and maintenance of pregnancy.

During the follicular phase of the cycle, progesterone levels remain low (0.2-1.5 ng/mL).[1,6,7] Following the LH surge and ovulation, luteal cells in the ruptured follicle produce progesterone in response to LH. During this luteal phase, progesterone rises rapidly to a maximum of 10-20 ng/mL at 5 to 7 days following ovulation. If conception does not occur, progesterone levels decrease during the last four days of the cycle due to the regression of the corpus luteum.[1,6-11] If conception occurs, the levels of progesterone are maintained at mid-luteal levels by the corpus luteum until about week six. At that time, the placenta becomes the main source of progesterone and levels rise from approximately 10-50 ng/mL in the first trimester to 50-280 ng/mL in the third trimester.[1,12,13]

Serum progesterone is a reliable indicator of either natural or induced ovulation because of its rapid rise following ovulation.[14-16] Disorders of ovulation, including anovulation, are relatively frequent and are responsible for infertility in approximately 15-20% of patients. Progesterone levels are abnormally low in these patients during the mid-luteal phase.

Luteal phase deficiency is a reproductive disorder associated with infertility and spontaneous abortion and is thought to occur in 10% of infertile women.[17-19] The infertility and pregnancy loss associated with this disorder are thought to be attributable to inadequate development of the endometrium.[20] The failure of the endometrium to mature is thought to be caused by insufficient production of progesterone by the corpus luteum. Progesterone levels in the luteal phase are lower than normal in women with luteal phase deficiency.[21,22]

Measurement of progesterone in the first 10 weeks of gestation has been shown to be reliable and effective for the diagnosis and treatment of patients with threatened abortion[23] and ectopic pregnancy. Suppressed progesterone levels (5 to 25 ng/mL) in the presence of detectable amounts of hCG is highly suggestive of patients with threatened abortion or ectopic pregnancy, regardless of gestational age.[24-26]

BIOLOGICAL PRINCIPLES OF THE PROCEDURE

The ARCHITECT Progesterone assay is a one-step immunoassay to determine the presence of progesterone in human serum and plasma using Chemiluminescent Microparticle Immunoassay (CMIA) technology with flexible assay protocols, referred to as Chemiflex.

Sample, anti-fluorescein (mouse, monoclonal) fluorescein-progesterone complex coated paramagnetic microparticles, and anti-progesterone (sheep, monoclonal) acridinium labeled conjugate are combined to create the reaction mixture.

Progesterone present in the sample competes with the anti-fluorescein (mouse, monoclonal) fluorescein-progesterone complex coated microparticles for binding with anti-progesterone (sheep, monoclonal) acridinium-labeled conjugate to form antibody-antigen-antibody complexes. After washing, Pre-Trigger and Trigger Solutions are then added to the reaction mixture; the resulting chemiluminescent reaction is measured as relative light units (RLUs). An inverse relationship exists between the amount of progesterone in the sample and the RLUs detected by the ARCHITECT *i* optical system.

For additional information on system and assay technology, refer to the ARCHITECT System Operations Manual, Section 3.

REAGENTS

Reagent Kit, 100 Tests

NOTE: Some kit sizes are not available in all countries or for use on all ARCHITECT *i* Systems. Please contact your local distributor.

ARCHITECT Progesterone Reagent Kit (7K77)

- **MICROPARTICLES** 1 or 4 Bottle(s) (6.6 mL) Anti-fluorescein (mouse, monoclonal) fluorescein progesterone complex coated Microparticles in TRIS buffer with protein (bovine and murine) and surfactant stabilizers. Concentration: 0.1% solids. Preservatives: sodium azide and ProClin.
- **CONJUGATE** 1 or 4 Bottle(s) (17.0 mL) Anti-progesterone (sheep, monoclonal) acridinium-labeled Conjugate in MES buffer with protein (bovine and sheep) stabilizers. Minimum concentration: 7 ng/mL. Preservatives: sodium azide and ProClin.
- **ASSAY DILUENT** 1 or 4 Bottle(s) (8.0 mL) Progesterone Assay Diluent contains TRIS buffer. Preservative: sodium azide.

Manual Diluent

ARCHITECT Progesterone Manual Diluent (7K77-50)

- **MANUAL DILUENT** 1 Bottle (5.0 mL) of ARCHITECT Progesterone Manual Diluent containing TRIS buffer. Preservative: sodium azide.

Other Reagents

ARCHITECT *i* Pre-Trigger Solution

- **PRE-TRIGGER SOLUTION** Pre-Trigger Solution containing 1.32% (w/v) hydrogen peroxide.

ARCHITECT *i* Trigger Solution

- **TRIGGER SOLUTION** Trigger Solution containing 0.35N sodium hydroxide.

ARCHITECT *i* Wash Buffer

NOTE: Bottle and volume varies based on order.

- **WASH BUFFER** Wash Buffer containing phosphate buffered saline solution. Preservatives: antimicrobial agents.

WARNINGS AND PRECAUTIONS

- **IVD**
- For *In Vitro* Diagnostic Use
- Package insert instructions must be carefully followed. Reliability of assay results cannot be guaranteed if there are any deviations from the instructions in this package insert.

Safety Precautions

- **CAUTION:** This product requires the handling of human specimens. It is recommended that all human sourced materials be considered potentially infectious and handled in accordance with the OSHA Standard on Bloodborne Pathogens.[27] Biosafety Level 2[28] or other appropriate biosafety practices[29,30] should be used for materials that contain or are suspected of containing infectious agents.

The following warnings and precautions apply to these components:

- Microparticles
- Conjugate

**WARNING:**	Contains methylisothiazolones.
H317	May cause an allergic skin reaction.
**Prevention**	
P261	Avoid breathing mist / vapours / spray.
P272	Contaminated work clothing should not be allowed out of the workplace.
P280	Wear protective gloves / protective clothing / eye protection.
**Response**	
P302+P352	IF ON SKIN: Wash with plenty of soap and water.
P333+P313	If skin irritation or rash occurs: Get medical advice / attention.
P363	Wash contaminated clothing before use.

This material and its container must be disposed of in a safe way.

- This product contains sodium azide. For a specific listing, refer to the **REAGENTS** section. Contact with acids liberates very toxic gas. This material and its container must be disposed of in a safe way.
- For information on the safe disposal of sodium azide and a detailed discussion of safety precautions during system operation, refer to the ARCHITECT System Operations Manual, Section 8.

Handling Precautions

- Do not use reagent kits beyond the expiration date.
- **Do not mix reagents from different reagent kits.**
- **The ARCHITECT Progesterone Reagent Kit must be maintained continuously at 2-8°C when not on-board the ARCHITECT *i* System. Performance differences may be seen if reagents are not at 2-8°C prior to loading them on the system.**
- Once the ARCHITECT Progesterone Reagent Kit has been removed from refrigerated storage (2-8°C), **immediately** place them on-board the ARCHITECT *i* System.
- Prior to loading the ARCHITECT Progesterone Reagent Kit on the system for the first time, the microparticle bottle requires mixing to resuspend microparticles that have settled during shipment. For microparticle mixing instructions, refer to the **Mixing Instructions** section below.
- **Septums MUST be used to prevent reagent evaporation and contamination and to ensure reagent integrity. Reliability of assay results cannot be guaranteed if septums are not used according to the instructions in this package insert.**
- To avoid contamination, wear clean gloves when placing a septum on an uncapped reagent bottle.
- Once a septum has been placed on an open reagent bottle, **do not invert the bottle** as this will result in reagent leakage and may compromise assay results.
- Over time, residual liquids may dry on the septum surface. These are typically dried salts which have no effect on assay efficacy.
- For a detailed discussion of handling precautions during system operation, refer to the ARCHITECT System Operations Manual, Section 7.

Mixing Instructions

Before loading the ARCHITECT Progesterone Reagent Kit on the system for the first time, the microparticle bottle requires mixing to resuspend microparticles that have settled during shipment:

- Invert the microparticle bottle 30 times.
- Visually inspect the bottle to ensure microparticles are resuspended. If microparticles are still adhered to the bottle, continue to invert the bottle until the microparticles have been completely resuspended.
- **If the microparticles do not resuspend, DO NOT USE. Contact your local Abbott representative.**

Storage Instructions

- 8°C 2°C The ARCHITECT Progesterone Reagent Kit must be stored at 2-8°C and may be used immediately after removal from 2-8°C storage.

> **NOTE:** The ARCHITECT Progesterone Reagent Kit is shipped cold and should be stored at 2-8°C after receipt. Refer to the **Handling Precautions** section in this package insert for additional information.

- When stored and handled as directed, reagents are stable until the expiration date.
- The ARCHITECT Progesterone Reagent Kit may be stored on-board the ARCHITECT *i* System for a maximum of 30 days. After 30 days, the reagent kit must be discarded. For information on tracking on-board time, refer to the ARCHITECT System Operations Manual, Section 5.
- Reagents may be stored on or off the ARCHITECT *i* System. If reagents are removed from the system, **immediately** store them at 2-8°C (with septums and replacement caps) in an upright position. For reagents stored off the system, it is recommended that they be stored in their original trays and boxes to ensure they remain upright. **If the microparticle bottle does not remain upright (with a septum installed) while in 2-8°C storage off the system, the reagent kit must be discarded.** After reagents are removed from the system, you must initiate a scan to update the on-board stability timer.

Indications of Reagent Deterioration

When a control value is out of the specified range, it may indicate deterioration of the reagents or errors in technique. Associated test results may be invalid and may require retesting. Assay recalibration may be necessary. For troubleshooting information, refer to the ARCHITECT System Operations Manual, Section 10.

INSTRUMENT PROCEDURE

- The ARCHITECT Progesterone assay file must be installed on the ARCHITECT *i* System from the ARCHITECT *i* Assay CD-ROM prior to performing the assay. For detailed information on assay file installation and on viewing and editing assay parameters, refer to the ARCHITECT System Operations Manual, Section 2.
- For information on printing assay parameters, refer to the ARCHITECT System Operations Manual, Section 5.
- For a detailed description of system procedures, refer to the ARCHITECT System Operations Manual.
- The default result unit for the ARCHITECT Progesterone assay is ng/mL. An alternate result unit, nmol/L, may be selected for reporting results by editing assay parameter “Result concentration units”, to nmol/L. The conversion factor used by the system is 3.18.

SPECIMEN COLLECTION AND PREPARATION FOR ANALYSIS

- Human serum (including serum collected in serum separator tubes) or plasma collected in sodium heparin, lithium heparin or potassium EDTA may be used in the ARCHITECT Progesterone assay. Other anticoagulants have not been validated for use with the ARCHITECT Progesterone assay. Follow the tube manufacturer’s processing instructions for serum or plasma collection tubes.
- Literature suggests that measurable progesterone may decrease with time when stored in serum separator tubes.[31] Serum collected in serum separator tubes and stored up to 24 hours on the gel showed (on average) a 13% loss.
- The ARCHITECT *i* System does not provide the capability to verify specimen type. It is the responsibility of the operator to verify the correct specimen types are used in the ARCHITECT Progesterone assay.
- Use caution when handling patient specimens to prevent cross contamination. Use of disposable pipettes or pipette tips is recommended.
- For optimal results, inspect all samples for bubbles. Remove bubbles with an applicator stick prior to analysis. Use a new applicator stick for each sample to prevent cross contamination.
- For optimal results, serum and plasma specimens should be free of fibrin, red blood cells, and other particulate matter.
- Ensure that complete clot formation in serum specimens has taken place prior to centrifugation. Some specimens, especially those from patients receiving anticoagulant or thrombolytic therapy may exhibit increased clotting time. If the specimen is centrifuged before a complete clot forms, the presence of fibrin may cause erroneous results.
- If testing will be delayed more than 24 hours, remove serum or plasma from the clot, serum separator, or red blood cells. Specimens may be stored for up to 10 days at 2-8°C prior to being tested. If testing will be delayed more than 10 days, specimens should be frozen at -10°C or colder. Specimens stored frozen at -10°C or colder for 6 months showed no performance difference.
- Multiple freeze-thaw cycles of specimens should be avoided. Specimens must be mixed THOROUGHLY after thawing, by LOW speed vortexing or by gently inverting, and centrifuged prior to use to remove red blood cells or particulate matter to ensure consistency in the results.
- Specimens with obvious microbial contamination should not be used.
- When shipped, specimens must be packaged and labeled in compliance with applicable state, federal and international regulations covering the transport of clinical specimens and infectious substances. Specimens must be shipped refrigerated under thermally controlled conditions or frozen (dry ice). Prior to shipment, it is recommended that specimens be removed from the clot, serum separator, or red blood cells.

PROCEDURE

Materials Provided

- 7K77 ARCHITECT Progesterone Reagent Kit

Materials Required But Not Provided

- ARCHITECT *i* System
- ARCHITECT *i* Assay CD-ROM
- 7K77-01 ARCHITECT Progesterone Calibrators
- 7K77-10 ARCHITECT Progesterone Controls
- 7K77-50 ARCHITECT Progesterone Manual Diluent
- ARCHITECT *i* PRE-TRIGGER SOLUTION
- ARCHITECT *i* TRIGGER SOLUTION
- ARCHITECT *i* WASH BUFFER
- ARCHITECT *i* REACTION VESSELS
- ARCHITECT *i* SAMPLE CUPS
- ARCHITECT *i* SEPTUM
- ARCHITECT *i* REPLACEMENT CAPS
- For information on materials required for maintenance procedures, refer to the ARCHITECT System Operations Manual, Section 9.
- Pipettes or pipette tips (optional) to deliver the volumes specified on the patient or control order screen.

Assay Procedure

- Order tests.
- Load the ARCHITECT Progesterone Reagent Kit on the ARCHITECT *i* System. Verify that all necessary assay reagents are present. Ensure that septums are present on all reagent bottles.
- The minimum sample cup volume is calculated by the system and is printed on the Orderlist report. No more than 10 replicates may be sampled from the same sample cup. To minimize the effects of evaporation verify adequate sample cup volume is present prior to running the test.
  - Priority: 100 µL for the first Progesterone test plus 50 µL for each additional Progesterone test from the same sample cup.
  - ≤ 3 hours onboard: 150 µL for the first Progesterone test plus 50 µL for each additional Progesterone test from the same sample cup.
  - > 3 hours onboard: additional sample volume is required. Refer to the ARCHITECT System Operations Manual, Section 5, for information on sample evaporation and volumes.
- If using primary or aliquot tubes, use the sample gauge to ensure sufficient patient specimen is present.
- ARCHITECT Progesterone Calibrators and Controls must be mixed THOROUGHLY by low speed vortex or inversion prior to use.
- To obtain the recommended volume requirements for the ARCHITECT Progesterone Calibrators and Controls, dispense a minimum of 200 µL of each calibrator or a minimum of 150 µL of each control into each respective sample cup.
- Load samples.
  - For information on loading samples, refer to the ARCHITECT System Operations Manual, Section 5.
- Press RUN. The ARCHITECT *i* System performs the following function:
  - Moves the sample to the aspiration point.
  - Loads a reaction vessel (RV) into the process path.
  - Aspirates and transfers sample into the RV.
  - Advances the RV one position and transfers microparticles and conjugate into the RV.
  - Mixes, incubates, and washes the reaction mixture.
  - Adds Pre-Trigger and Trigger Solutions.
  - Measures chemiluminescent emission to determine the quantity of progesterone in the sample.
  - Aspirates contents of RV to liquid waste and unloads RV to solid waste.
  - Calculates the result.
- For information on ordering patient specimens, calibrators and controls, and general operating procedures, refer to the ARCHITECT System Operations Manual, Section 5.
- For optimal performance, it is important to follow the routine maintenance procedures defined in the ARCHITECT System Operations Manual, Section 9. If your laboratory requires more frequent maintenance, follow those procedures.

Specimen Dilution Procedures

Specimens with a progesterone value exceeding 40 ng/mL are flagged with the code " > 40" and may be diluted using either the Automated Dilution Protocol or the Manual Dilution Procedure.

Automated Dilution Procedure

- If using the Automated Dilution Protocol, the system performs a 1:10 dilution of the specimen and automatically calculates the concentration of the specimen before dilution and reports the result.

Manual Dilution Procedure

- The suggested dilution for Progesterone is 1:10. It is recommended dilutions not exceed 1:15.
- For a 1:10 dilution, add 50 µL of the patient specimen to 450 µL of ARCHITECT Progesterone Manual Diluent (7K77-50).
- The operator must enter the dilution factor in the patient or control order screen. The system will use this dilution factor to automatically calculate the concentration of the specimen before dilution. This will be the reported result. The dilution should be performed so that the diluted result reads greater than 10.0 ng/mL for a 1:10 dilution.
- If the operator does not enter the dilution factor, the reported result will be that of the diluted sample. This result (before dilution factor is applied) should be greater than 1.0 ng/mL. **The reported result must be multiplied by the dilution factor to obtain the concentration of the undiluted sample.**

For detailed information on ordering dilutions, refer to the ARCHITECT System Operations Manual, Section 5.

Calibration

- To perform an ARCHITECT Progesterone calibration, test Calibrators 1 and 2 in duplicate. A single sample of all levels of progesterone controls must be tested to evaluate the assay calibration. Ensure that assay control values are within the concentration ranges specified in the control package insert. Calibrators should be priority loaded.
- Calibration Range: 0 - 40 ng/mL.
- Calibration Frequency

  Once an ARCHITECT Progesterone calibration is accepted and stored, all subsequent samples may be tested without further calibration unless:
  - A reagent kit with a new lot number is used.
  - Controls are out of package insert control ranges.

  For best results,
  - Establish statistically-based QC ranges to monitor and control the frequency of recalibration, or
  - Establish a 30-day limit of recalibration frequency to optimize the performance of your assay.
- For detailed information on how to perform an assay calibration, refer to the ARCHITECT System Operations Manual, Section 6.

QUALITY CONTROL PROCEDURES

The recommended control requirement for the ARCHITECT Progesterone assay is a single sample of all control levels tested once every 24 hours each day of use. If the quality control procedures in your laboratory require more frequent use of controls to verify test results, follow your laboratory-specific procedures. Ensure that assay control values are within the concentration ranges specified in the control package insert.

Verification of Assay Claims

For protocols to verify package insert claims, refer to the ARCHITECT System Operations Manual, Appendix B. The ARCHITECT Progesterone assay belongs to method group 1.

RESULTS

The ARCHITECT Progesterone assay utilizes a 4 Parameter Logistic Curve Fit data reduction method (4PLC, Y weighted) to generate a calibration curve.

Alternate Result Units

- The default result unit for the ARCHITECT Progesterone assay is ng/mL. When the alternate result unit, nmol/L, is selected, the conversion factor used by the system is 3.18.
- Conversion Formula: (Concentration in ng/mL) x (3.18) = nmol/L

Flags

- Some results may contain information in the Flags field. For a description of the flags that may appear in this field, refer to the ARCHITECT System Operations Manual, Section 5.

LIMITATIONS OF THE PROCEDURE

- For diagnostic purposes, results should be used in conjunction with other data; e.g., symptoms, results of other tests, clinical impressions, etc.
- If the progesterone results are inconsistent with clinical evidence, additional testing is suggested to confirm the results.
- Specimens from patients who have received preparations of mouse monoclonal antibodies for diagnosis or therapy may contain human anti-mouse antibodies (HAMA). Such specimens may show either falsely elevated or depressed values when tested with assay kits which employ mouse monoclonal antibodies.[32,33] Additional information may be required for diagnosis.
- Heterophilic antibodies in human serum can react with reagent immunoglobulins, interfering with *in vitro* immunoassays.[34] Patients routinely exposed to animals or to animal serum products can be prone to this interference and anomalous values may be observed. Additional information may be required for diagnosis.

EXPECTED VALUES

The expected ranges for the ARCHITECT Progesterone assay were obtained by testing specimens drawn from 63 males, 36 postmenopausal females, 20 normal menstruating females, and from 100 females in the first, second, or third trimester of pregnancy. For this study, specimens from normal menstruating females were categorized as follicular phase and luteal phase. Follicular phase was defined as the period of time from 10 days to 5 days prior to the day in which LH and FSH were most elevated. The luteal phase was defined as the period of time from 4 days to 10 days after the day on which LH and FSH were most elevated.

The results are presented below.

Population	n	Progesterone Value (ng/mL) Median	Range
Normal Menstruating Females:			
Follicular Phase	91	0.1	< 0.1 - 0.3
Luteal Phase	60	8.5	1.2 - 15.9*
Postmenopausal Females:	36	0.1	< 0.1 - 0.2
Pregnant Females:			
First Trimester	35	20.9	2.8 - 147.3
Second Trimester	27	45.4	22.5 - 95.3
Third Trimester	38	87.4	27.9 - 242.5
Males:	63	< 0.1	< 0.1 - 0.2

* Luteal phase represents the central 95% interval of all values.

It is recommended that each laboratory establish its own expected ranges.

SPECIFIC PERFORMANCE CHARACTERISTICS

Precision

The ARCHITECT Progesterone assay is designed to have a precision of ≤ 10% total CV for concentrations in the range of the ARCHITECT Progesterone Low Control and ≤ 7% total CV for concentrations in the ranges of the ARCHITECT Progesterone Medium and High Controls.

Precision was determined as described in the Clinical and Laboratory Standards Institute (CLSI, formerly NCCLS) Protocol EP5-T2.[35] A three member buffered protein based panel was assayed, using two lots of reagents, in replicates of two at two separate times per day for 20 days on two instruments. Data from this study are summarized below.*

Panel Member	Reagent Lot	Instrument	n	Mean Conc. Value (ng/mL)	Within Run SD	Within Run %CV	Total SD	Total %CV
1	1	1	80	0.8	0.046	5.5	0.052	6.2
1	1	2	80	0.8	0.045	5.4	0.048	5.8
1	2	1	80	0.8	0.027	3.4	0.038	4.7
1	2	2	80	0.8	0.037	4.7	0.044	5.6
2	1	1	80	4.8	0.073	1.5	0.101	2.1
2	1	2	80	4.7	0.111	2.4	0.135	2.9
2	2	1	80	4.7	0.097	2.1	0.111	2.4
2	2	2	80	4.5	0.082	1.8	0.129	2.8
3	1	1	80	21.2	0.340	1.6	0.445	2.1
3	1	2	80	21.1	0.459	2.2	0.542	2.6
3	2	1	80	21.0	0.400	1.9	0.529	2.5
3	2	2	80	20.4	0.374	1.8	0.805	3.9

* Representative data; results in individual laboratories may vary from these data.

Recovery

The ARCHITECT Progesterone assay is designed to have a mean recovery of 90% to 110%, inclusive.

Known concentrations of progesterone were added to five aliquots of human serum. The concentration of progesterone was determined using the ARCHITECT Progesterone assay. The percent recovery of the ARCHITECT Progesterone assay ranged from 90.0% to 107.0% with a mean of 96.4%.

Analytical Sensitivity

The ARCHITECT Progesterone assay is designed to have an analytical sensitivity of ≤ 0.1 ng/mL.

The analytical sensitivity of the ARCHITECT Progesterone assay was calculated to be better than 0.1 ng/mL (n = 36 runs). Analytical sensitivity is defined as the concentration at two standard deviations from the mean RLU value of the ARCHITECT Progesterone MasterCheck Level 0 (0.0 ng/mL), and represents the lowest measurable concentration of progesterone that can be distinguished from zero.

Specificity

The specificity of the ARCHITECT Progesterone assay was determined by studying the cross reactivity of the compounds listed below. Human serum specimens containing essentially no residual progesterone were supplemented with potential cross reactants at the concentrations listed and tested for progesterone. Cross reactivity is stated below.

Cross Reactant	Cross Reactant Concentration (ng/mL)	% Cross Reactivity
Corticosterone	1000	4.6
Danazol	1000	0.1
11-Deoxycorticosterone	1000	1.8
20 α-hydroxyprogesterone	1000	0.2
20 β-hydroxyprogesterone	1000	0.3
17-Hydroxyprogesterone	1000	2.9
Medroxyprogesterone	1000	0.1
19-Nor-4-androsten-3, 17-dione	1000	0.1
Norethindrone	1000	0.1
19-Nortestosterone	1000	0.1
5 α-Pregnan-3, 20-dione	1000	3.3
5 α-Pregnan-3 α-ol-20-one	1000	0.9
5 α-Pregnan-3 β-ol-20-one	1000	0.3
5 Pregnan-3-ol-20-one	1000	3.9
Pregnanolone	1000	1.3
Pregnenolone	1000	0.1
Testosterone	1000	0.2

Cross reactivity of the following compounds was undetectable.

Cross Reactant	Cross Reactant Concentration (ng/mL)
Aldosterone	1000
Allopregnanediol	1000
Androstenediol	1000
Androstenedione	1000
Clomiphene Citrate	1000
Cortisol	1000
11-Deoxycortisol	1000
Desogestrel	1000
DHEA	1000
DHEA-S	100000
Dihydrotestosterone	1000
Estradiol (17β)	1000
Estriol	1000
Estrone	1000
Ethisterone	1000
Ethynyl-Estradiol	1000
Ethynodiol diacetate	1000
17-Hydroxypregnenolone	1000
Medroxyprogesterone Acetate	1000
Methylprednisolone	1000
Norethindrone Acetate	1000
Norgestrel	1000
Normethandrone	1000
5 β-Pregnane	1000
5 β-Pregnan-3 α, 20 α-diol	1000
Pregnenolone 3 Sulfate	1000
Spironolactone	1000

Interference

Potential interference from hemoglobin, bilirubin, triglycerides, and protein was studied in the ARCHITECT Progesterone assay. The ARCHITECT Progesterone assay demonstrated the interference stated below.

- Hemoglobin < 10% at 500 mg/dL
- Bilirubin < 10% at 20 mg/dL
- Triglycerides < 10% at 1000 mg/dL
- Protein < 10% at 4 g/dL and 12 g/dL

Accuracy by Correlation

The ARCHITECT Progesterone assay is designed to have a slope of 0.8 to 1.2, inclusive, and a correlation coefficient of ≥ 0.95 when compared to a commercially available assay.

The ARCHITECT Progesterone assay was compared to a commercially available diagnostic kit. The results of the specimen testing are shown below.‡

Abbott ARCHITECT Progesterone vs. commercially available diagnostic kit

Method	Number of Specimens	Intercept	Slope	Correlation Coefficient
Least Squares Linear Regression	199	-0.4	0.81	0.990
Passing-Bablok Linear Regression*	199	-0.4	0.83	0.990

‡ Representative data: variables such as differences in sampling size and sample population may impact the correlation of the assay; therefore, results in individual laboratories may vary from these data.

In this evaluation, serum samples tested ranged from 0.1 ng/mL to 36.0 ng/mL with the ARCHITECT Progesterone assay.

* A linear regression method with no special assumptions regarding the distribution of the samples and the measurement errors.[36]

BIBLIOGRAPHY

1. Abraham GE, Odell WD, Swerdloff RS, Hopper K. Simultaneous radioimmunoassay of plasma FSH, LH, progesterone, 17-hydroxyprogesterone, and estradiol-17β during the menstrual cycle. *J Clin Endocr*. 1972;34:312-318.
2. Strauss JF III, Hsueh AJW. Ovarian hormone synthesis. In: DeGroot LJ, Jameson JL, *et al.* eds. *Endocrinology*. Vol 3. 4th ed. Philadelphia: WB Saunders Co., 2001. 2043-2052.
3. Weigel NL, Rowan BG. Estrogen and progesterone action. In: DeGroot LJ, Jameson JL, *et al.* eds. *Endocrinology*. Vol 3. 4th ed. Philadelphia: WB Saunders Co., 2001. 2053-2060.
4. Erickson GF. Folliculogenesis, ovulation, and luteogenesis. In: DeGroot LJ, Jameson JL, *et al.* eds. *Endocrinology*. Vol 3. 4th ed. Philadelphia: WB Saunders Co., 2001. 2061-2071.
5. Hertig AT, Livingstone RG. Spontaneous, threatened and habitual abortion: their pathogenesis and treatment. *N Eng J Med*. 1944;230: 797-806.
6. Aedo AR, Nuñez M, Landgren B-M, *et al.* Studies on the pattern of circulating steroids in the normal menstrual cycle. *Acta Endocrinol*. 1977;84:320-332.
7. Landgren B-M, Undén A-L, Diczfalusy E. Hormonal profile of the cycle in 68 normally menstruating women. *Acta Endocrinol*. 1980;94:89-98.
8. Erickson GF. Normal ovarian function. *Clin Obstet Gynecol*. 1978; 21:31-52.
9. Veldhuis JD, Christiansen E, Evans WS, *et al.* Physiological profiles of episodic progesterone release during the midluteal phase of the human menstrual cycle: analysis of circadian and ultradian rhythms, discrete pulse properties, and correlations with simultaneous luteinizing hormone release. *J Clin Endocrinol Metab*. 1988;66:414-421.
10. Filicori M, Butler JP, Crowley WF Jr. Neuroendocrine regulation of the corpus luteum in the human. *J Clin Invest*. 1984;73:1638-1647.
11. Laufer N, Navot D, Schenker JG. The pattern of luteal phase plasma progesterone and estradiol in fertile cycles. *Am J Obstet Gynecol*. 1982;143:808-813.
12. Winkel P, Gaede P, Lyngbye J. Method for monitoring plasma progesterone concentrations in pregnancy. *Clin Chem*. 1976;22: 422-428.
13. Buster JE, Abraham GE. The applications of steroid hormone radioimmunoassays to clinical obstetrics. *Obstet Gynecol*. 1975;46: 489-499.
14. Israel R, Mishell DR, Stone SC, *et al.* Single luteal phase serum progesterone assay as an indicator of ovulation. *Am J Obstet Gynecol*. 1972;112:1043-1046.
15. Petsos P, Chandler C, Oak M, *et al*. The assessment of ovulation by a combination of ultrasound and detailed serial hormone profiles in 35 women with long-standing unexplained infertility. *Clin Endocrinol*. 1985;22:739-751.
16. Abdulla U, Diver MJ, Hipkin LJ, Davis JC. Plasma progesterone levels as an index of ovulation. *Br J Obstet Gynaecol*. 1983;90:543-548.
17. Rosenberg SM, Luciano AA, Riddick DH. The luteal phase defect: the relative frequency of, and encouraging response to, treatment with vaginal progesterone. *Fertil Steril*. 1980;34:17-20.
18. Tho PT, Byrd JR, McDonough PG. Etiologies and subsequent reproductive performance of 100 couples with recurrent abortion. *Fertil Steril*. 1979;32:389-395.
19. Hernández Horta JL, Gordillo Fernández J, Soto de León B, Cortés-Gallegos V. Direct evidence of luteal insufficiency in women with habitual abortion. *Obstet Gynecol*. 1977;49:705-708.
20. Jones GS. The physiology of menstruation and the corpus luteum function. *Int J Fertil*. 1986;31:143-147.
21. Soules MR, McLachlan RI, Ek M, *et al.* Luteal phase deficiency: characterization of reproductive hormones over the menstrual cycle. *J Clin Endocrinol Metab*. 1989;69:804-812.
22. Schweiger U, Laessle R, Schweiger M, *et al*. Caloric intake, stress and menstrual function in athletes. *Fertil Steril*. 1988;49:447-450.
23. Witt BR, Wolf GC, Wainwright CJ, *et al.* Relaxin, CA-125, progesterone, estradiol, Schwangerschaft protein, and human chorionic gonadotropin as predictors of outcome in threatened and nonthreatened pregnancies. *Fertil Steril*. 1990;53:1029-1036.

24. Matthew CP, Coulson PB, Wild RA. Serum progesterone levels as an aid in the diagnosis of ectopic pregnancy. *Obstet Gynecol*. 1986;68:390-394.
25. Hubinont CJ, Thomas C, Schwers JF. Luteal function in ectopic pregnancy. *Am J Obstet Gynecol*. 1987;156:669-674.
26. Wallach J. *Interpretation of Diagnostic Tests*. 7th ed. Philadelphia: Lippincott Williams & Wilkins, 2000:761-763.
27. US Department of Labor, Occupational Safety and Health Administration, 29 CFR Part 1910.1030, Bloodborne pathogens.
28. US Department of Health and Human Services. *Biosafety in Microbiological and Biomedical Laboratories.* 5th ed. Washington, DC: US Government Printing Office; January 2007.
29. World Health Organization. *Laboratory Biosafety Manual.* 3rd ed. Geneva: World Health Organization; 2004.
30. Clinical and Laboratory Standards Institute. *Protection of Laboratory Workers from Occupationally Acquired Infections: Approved Guideline—Third Edition.* CLSI Document M29-A3. Wayne, PA: Clinical and Laboratory Standards Institute; 2005.
31. Wild D, ed. *The Immunoassay Handbook*. 2nd ed. London: Nature Publishing Group, 2001:418.
32. Primus FJ, Kelley EA, Hansen HJ, Goldenberg DM. “Sandwich ”-type immunoassay of carcinoembryonic antigen in patients receiving murine monoclonal antibodies for diagnosis and therapy. *Clin Chem*. 1988;34:261-264.
33. Schroff RW, Foon KA, Beatty SM, *et al.* Human anti-murine immunoglobulin responses in patients receiving monoclonal antibody therapy. *Cancer Res*. 1985;45:879-885.
34. Boscato LM, Stuart MC. Heterophilic antibodies: a problem for all immunoassays. *Clin Chem*. 1988;34:27-33.
35. National Committee for Clinical Laboratory Standards. *Evaluation of Precision Performance of Clinical Chemistry Devices - Second Edition; Tentative Guideline*. NCCLS Document EP5-T2. Villanova, PA: NCCLS, March 1992.
36. Passing H, Bablok W. A new biometrical procedure for testing the equality of measurements from two different analytical methods. *J Clin Chem Clin Biochem*. 1983;21:709-720.

ARCHITECT, Chemiflex and MasterCheck are trademarks of Abbott Laboratories in various jurisdictions.
ProClin is property of its respective owner.

Abbott Ireland
Diagnostics Division
Lisnamuck, Longford
Co. Longford
Ireland
+353-43-3331000

Distributed by Abbott Laboratories
Abbott Park, IL 60064 USA
and
ABBOTT 65205 Wiesbaden, Germany

April 2010
© 2006, 2010 Abbott Laboratories
